# Spatiotemporal distribution and risk assessment of bisphenol A and structurally related phenolic compounds in groundwater around the vicinity of municipal dumpsites in Southwestern Nigeria

**DOI:** 10.1039/d5ra07962d

**Published:** 2026-02-13

**Authors:** Esther A. Nnamani, Ajibola A. Bayode, Moses O. Alfred, Brigitte Helmreich, Emmanuel I. Unuabonah, Martins O. Omorogie

**Affiliations:** a African Centre of Excellence for Water and Environmental Research (ACEWATER), Redeemer's University PMB 230 Ede Nigeria; b Department of Chemical Sciences, Redeemer's University PMB 230 Ede Nigeria; c Chair of Urban Water Systems Engineering, School of Engineering and Design, Technical University of Munich Am Coulombwall 3 Garching D-85748 Germany mo.omorogie@tum.de omorogiem@run.edu.ng dromorogiemoon@gmail.com

## Abstract

Leachate infiltration into groundwater is an intensifying environmental and public health concern in many developing countries, where waste disposal systems are poorly engineered. Phenolic contaminants pose significant ecological and human health risks but remain understudied in sub-Saharan Africa. This study investigated the seasonal occurrence, distribution, and associated risks of four phenolic compounds, namely bisphenol A (BPA), hydroquinone (HQ), resorcinol (RE), and benzoquinone (BQ), in groundwater sources near municipal dumpsites across three Southwestern States in Nigeria. Groundwater samples were collected from rural and urban areas across Osun, Oyo, and Lagos States during both rainy and dry seasons. Solid-phase extraction and high-performance liquid chromatography-ultraviolet detector (HPLC-UV) analysis were used to extract and quantify the target analytes, followed by multivariate statistical evaluation, human health risk assessment, and ecological risk assessment. Concentrations of BPA, HQ, RE, and BQ were generally higher during the dry season, especially in Osun and Lagos States. BPA recorded the highest values, peaking at 20.90 mg L^−1^ in urban Osun. RE and BQ also showed elevated levels during the dry season, particularly in urban Lagos and rural Oyo, respectively. HQ exhibited variable trends, with significant peaks in rural Oyo (11.93 mg L^−1^) and rural Osun (7.26 mg L^−1^). Multivariate analysis *via* principal component revealed clear seasonal differentiation and strong co-loading patterns consistent with leachate-driven contamination processes. Human health risk assessment indicated that estimated daily intakes in children frequently exceeded stipulated limits, while ecological risk assessment identified *Daphnia magna* as the most sensitive species, with acute and chronic risk quotients exceeding the reference dose in several locations. This study represents the first year-long, multi-state assessment of phenolic contaminants in Nigerian groundwater and provides critical evidence of contamination levels linked to unmanaged waste sites. These findings highlight the urgent need for improved waste management, groundwater protection policies, and expanded toxicological evaluation of phenolic contaminants in rapidly urbanizing regions of sub-Saharan Africa.

## Abstract 2

Leachate infiltration into groundwater is an intensifying environmental and public health concern in many developing countries, where waste disposal systems are poorly engineered.

## Graphical abstract

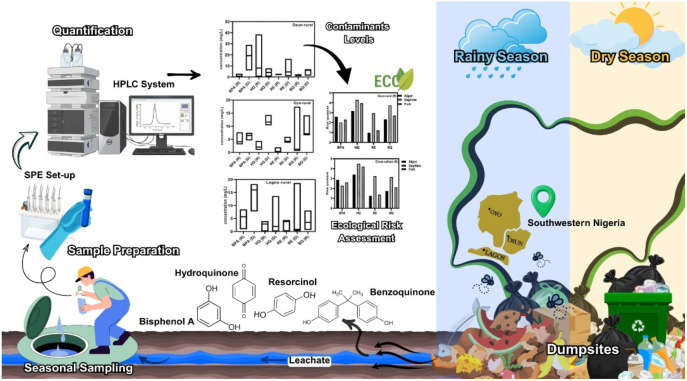

## 1 Introduction

Poor water quality accounts for 80% of disease and 50% of child mortality globally, underscoring the urgent need for safe and clean water as a fundamental human right.^1,2^ In sub-Saharan Africa, groundwater represents a predominant drinking-water source, supporting nearly 75% of the population.^3^ According to the United Nations Water Development Report (2022), groundwater accounts for approximately 99% of freshwater and plays a vital role in drinking, irrigation, and industrial activities.^4–6^ Although natural geological formations can provide some protection, groundwater remains vulnerable to irreversible contamination from anthropogenic sources, thereby posing significant public health concerns.^7,8^

Among several anthropogenic direct and indirect sources of groundwater contamination, endocrine-disrupting compounds (EDCs) are becoming increasingly alarming because of their ability to mimic the endocrine gland, disrupt its normal functioning, and trigger serious neurological, reproductive, and developmental issues in humans and aquatic organisms.^9^ Prominent in this class of EDCs are phenolic compounds. According to the United States Environmental Protection Agency (USEPA), phenolic compounds (PCs) have been classified as priority and hazardous pollutants because they have been noted to be toxic and have acute to chronic effects on humans and aquatic organisms.^10^ Phenolic compounds are quite distinct, and over 60 PCs have been quantified in surface water, ground water, urban run-off, rainwater, and packaged water globally.^7,11–15^ From these findings, many exceeded the regulatory limit up to millions of ng L^−1^ in some regions, to mg L^−1^.^16^

One of the primary environmental pathways through which PCs enter groundwater is leachate infiltration from landfills and open dumpsites, which are common waste disposal methods in many developing countries.^17,18^ These sites release a complex mixture of organic and inorganic contaminants that can degrade into environmentally hazardous compounds, including Bisphenol A (BPA), hydroquinone (HQ), benzoquinone (BQ), and resorcinol (RE).

In particular, BPA, a prevalent parent compound, can undergo photodegradation or microbial transformation, producing hydroquinone, which auto-oxidizes into benzoquinone and its isomer resorcinol.^19,20^ BPA is widely used in the production of polycarbonate plastics and epoxy resins.^21^ Hydroquinone, a highly water-soluble reducing agent, is used in the production of skin-lightening ointment. It is also used as a polymer stabilizer in photographic development as well as in the production of paints, varnishes, and other industrial lubricants.^22,23^ Benzoquinone is formed through the auto-oxidation of HQ. It is largely used as an oxidizing agent and in the synthesis of dyes and pesticides.^24^ While resorcinol is used in the pharmaceutical industry and in the production of resins, pigments, ultraviolet absorbers, adhesives, and rubber.^25^ Their diverse use across industries has increased their levels in groundwater systems and worsened human exposure to this class of EDCs.

These chemicals are highly concerning due to their multi-system toxicity, with risks ranging from respiratory disorders, neurological breakdown, morphological deformation, generation of reactive oxygen species (ROS), and potentially the growth of cancerous cells.^7,26^ Human exposure to these compounds can occur predominantly through ingestion, inhalation, and dermal absorption.^27^ Literature confirms that pre-natal exposure to BPA, even at trace concentrations, can affect the normal functioning of the brain and liver, and may induce hepato-carcinogenesis in the liver.^28^ Beyond their toxicity, these PCs are noted for their high environmental persistence due to their varying octanol–water partition coefficient, which influences their mobility in both organic matter and aqueous media.^29^

Given the toxicity and persistence of these byproducts, understanding their occurrence and pollution profiles in groundwater systems is integral to assessing eco-human risks and informing water safety interventions. It is rather disheartening that there are yet to be established stipulated limits by the approved and appropriate regulatory bodies globally for all the targeted analytes in this study, except for BPA, which was newly introduced and set at 0.0025 mg L^−1^ in drinking water by the World Health Organization (WHO) and the European Food Safety Authority (EFSA). This new limit is set to be implemented by January 2026.^30^

Furthermore, there are very few studies on the distribution and potential eco-human health risks of BPA, HQ, RE, and BQ in groundwater sources near dumpsites globally.^31^ Specifically, there is a lack of published studies on the occurrence, distribution, and risk assessment of RE and BQ in African groundwater sources. While global research emphasizes the removal of these compounds due to their toxicity, regional data, particularly in Africa, remain scarce.^22,32–34^ This represents a significant research gap because of the unregulated use and disposal of these compounds in developing regions where public health awareness and water purification techniques remain inadequate.

To address this critical deficiency, the study aims to comprehensively profile these compounds in groundwater, including borehole and hand-dug wells, and to evaluate ecological and human health risk assessments across multiple states using established toxicological benchmarks. The study also intends to contribute to the achievement of the United Nations Sustainable Development Goal 6 (Clean Water and Sanitation) by 2030. The targeted analytes (BPA, HQ, RE, and BQ) were selected based on their widespread industrial use, environmental persistence, and high leaching potential from unlined dumpsites into shallow aquifers. As hazardous phenolic compounds, they pose significant ecological and human health risks, especially in communities reliant on untreated groundwater. To the best of our knowledge, this study presents the first comprehensive evaluation of BPA, HQ, RE, and BQ in groundwater sources located near open dumpsites in Africa. It is hypothesized that the proximity of groundwater to uncontrolled waste disposal sites significantly elevates phenolic contaminant levels, thereby increasing potential risks to both human and ecological receptors. The findings establish an essential baseline for developing a targeted risk assessment framework. The physicochemical properties of the studied analytes are presented in Table SI 1.

## 2 Research methodology

### 2.1 Chemicals and standards

The PCs, namely, 4,4′-(propane-2,2-diyl) diphenol (bisphenol A), benzene-1,4-diol (hydroquinone), cyclohexa-2,5-diene-1,4-dione (benzoquinone), and benzene-1,3-diol (resorcinol), along with HPLC-grade methanol, Supel-Select Hydrophilic-lipophilic (HLB) solid phase extraction (200 mg, 6 mL), analytical-grade hydrochloric acid, acetone and sodium hydroxide were purchased from Sigma-Aldrich (St. Louis, MO, USA). All chemicals had ≥99.9% purity and were suitable for high-performance liquid chromatography (HPLC) applications. Ultrapure Millipore water was purchased from Fisher Scientific Standard (Germany). Standard stock solutions of individual PC (100 mg L^−1^) were prepared in methanol and preserved at 4 °C. A mixed working solution of 1 mg L^−1^ mixed PCs was freshly prepared by serial dilution with Millipore water and used in the matrix spiking procedure for method validation.

### 2.2 Sampling area and design

**Fig. 1:**
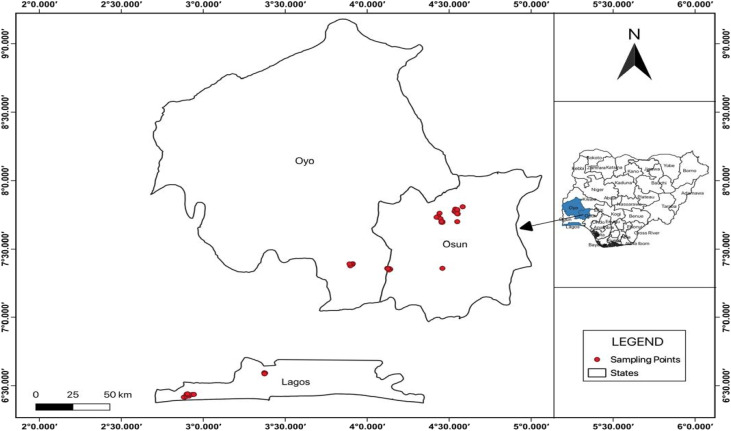
Map of the sampling sites in Osun, Oyo, and Lagos States, Southwestern Nigeria.

The study was conducted across both rural and urban areas in Osun, Oyo, and Lagos States, Nigeria, during the rainy and dry seasons. Osun, which has the fewest urban centers of the three, is predominantly agricultural and renowned for farming of crops such as maize, cassava, and millet. Oyo State, home to Ibadan, Nigeria's third-largest metropolitan area, is a hybrid landscape with significant civil-service employment, agro-processing, and light manufacturing industries. Lagos, the nation's premier industrial and commercial hub, is a densely urbanized metropolitan state with extensive industrial activity, heavy commercial development, and large-scale landfills such as Olusosun. The three states are located in the southwest of Nigeria. Osun and Oyo are the closest neighbours to Lagos, sharing regional watershed and geological characteristics, yet they differ markedly in urban density, economic structure, and potential sources of phenolic pollution. Dumpsites proximate to groundwater sources in residential areas were identified in both rural and urban settings of the three states. For each location, 10 sampling sites were selected, distributed across distance bands ranging from 50 m to 500 m from the edge of the dumpsite. At each site, three groundwater samples were collected as spatial replicates using 500 mL amber glass bottles, resulting in a total of 60 groundwater samples per season (rainy and dry). Samples were stored in ice packs while in the field and promptly transported to the laboratory to be preserved at 4 °C until extraction. Groundwater sampling was carried out during the rainy and dry seasons between July 2024 and May 2025. Physicochemical parameters, such as pH, total dissolved solids (TDS), and electric conductivity (EC), were measured *in situ* using a well-calibrated portable multiparameter metre (HANNA pH/EC/TDS HI 9811-5). The geographic coordinates (Table SI 2) were used to generate the environmental map (Fig. 1) of the sampled locations.

### 2.3 Sample preparation and extraction protocol

Groundwater samples were vacuum-filtered using a 0.22 µm cellulose ester filter membrane before analysis. The pH of the spiked samples and blank solutions was adjusted to 3.0 using 0.1 M HCl and 0.1 M NaOH to ensure optimal extraction efficiency. Solid-phase extraction (SPE) technique was performed using pre-conditioned HLB SPE cartridges (5 mL of methanol and ultrapure Millipore water each). Samples were loaded at a flow rate of 8.5 mL min^−1^. Subsequently, the cartridges were washed with 3 mL of ultrapure water, vacuum-dried for 5 min, and eluted with 5 mL of HPLC-grade methanol. The eluents were evaporated to near-dryness in a vacuum oven at 50 °C and reconstituted with 0.5 mL of acidified methanol before instrumental analysis. This procedure resulted in a pre-concentration factor of 500, enhancing detection sensitivity and ensuring accurate quantification of the target analytes.

### 2.4 Instrumentation technique

High-Performance Liquid Chromatography-Ultraviolet Detector (HPLC-UV) of Agilent Series 1100 LC system (Agilent Technologies, Germany) was used for both the quantitative and qualitative analysis of the studied analytes. Chromatographic separation was done with a reversed-phase C-18 Discovery ® HS (Cat#: 568523-U; 25 cm × 4.6 mm, 5 µm particle size) under isocratic elution with a mobile phase consisting of methanol/water (30/70, v/v) at a flow rate of 1 mL min^−1^. The column temperature was set at 30 °C. An autosampler was used to inject 20 µL of each sample with a total run time of 12 min. UV detection was set at 280 nm, which corresponds to the absorbance maxima of the phenolic PCs analyzed. Identification and quantification of the PCs were based on retention time and peak area integration according to the established calibration curve for each compound.

### 2.5 Quality control and quality assessment

Glassware was washed with distilled water and acetone, dried appropriately before use. The water samples were collected in amber glass bottles and transported to the laboratory in ice packs. An analysis of procedural blanks, spiked blanks, method blanks, and field blanks was carried out to monitor any form of contamination in the extraction process. Similarly, instrumental (methanol) blanks were analyzed to check for any cross-contamination of the analytes and any drift in instrument response after each run. To evaluate matrix effects and method recovery, 250 mL of each groundwater sample was spiked with 1 mg L^−1^ of mixed PCs (BPA, HQ, RE, and BQ) and processed in parallel with unspiked samples. Furthermore, matrix spike samples at 1 mg L^−1^ (low), 10 mg L^−1^ (medium), and 20 mg L^−1^ (high) were used to evaluate precision and reproducibility across the analytical range. Spiked samples were processed in parallel with unspiked environmental samples. Spiked samples were used solely for quality control, while environmental concentrations were reported exclusively from unspiked samples quantified using external calibration. Absolute recoveries of the targeted analytes were also assessed to evaluate the extraction efficiency and verify the suitability of the optimized analytical method. Matrix spike recoveries were consistently high (95–102%) with low RSDs, confirming excellent method accuracy and minimal matrix interference. Analytes were quantified using external standards, and a calibration curve was plotted by analyzing standard solutions with concentrations ranging from 0.1 mg L^−1^ to 20 mg L^−1^. The regression coefficient (*R*^2^) values for all the analytes were greater than 0.99, as shown in Table 1. Method detection limits (MDLs) were determined by analyzing seven replicate low-concentration standard solutions of the targeted analyte. MDL was calculated as the standard deviation of the replicates multiplied by the student's *t*-value at 99% confidence. The instrumental limit of detection (LOD) was calculated as three times the standard deviation of the regression intercepts (*σ*) divided by the slope (*S*) of a seven-point calibration curve (LOD = 3*σ*/*S*). The limit of quantification (LOQ) was defined as ten times this ratio (LOQ = 10*σ*/*S*), in line with established analytical protocols. Precision was assessed by analyzing triplicate spiked groundwater samples at three pre-determined concentration levels (low, medium, high) on the same day (intra-day) and across three consecutive days (inter-day). This quality control measure confirmed the reproducibility of the analytical technique. Results were expressed as % RSD of the measured concentrations, and were all below 12%, affirming consistent analytical performance.

**Table 1:** Analytical performance and precision of phenolic compounds in groundwater samples

Analyte	Linear range (mg L^−1^)	*R* ^2^	LOD^c^ (mg L^−1^)	LOQ^c^ (mg L^−1^)	MDL^c^ (mg L^−1^)	Field blank (mg L^−1^)	Method blank (mg L^−1^)	Spiked concentration (mg L^−1^)	Intra-day precision		Inter-day precision	
									Recovery (%) ± SD	RSD (%) *n* = 3^a^	Recovery (%) ± SD	RSD (%) *n* = 9^b^
BPA	0.1–20	0.9983	0.33	1.08	0.25	<LOD	<LOD	1.0	102 ± 5.3	5.0	100.6 ± 6.3	6.5
								10.0	102 ± 3.2	3.1	99.9 ± 4.0	4.0
								20.0	97.1 ± 2.3	2.4	96.8 ± 3.1	3.2
HQ	0.1–20	0.9981	0.34	1.13	0.26	<LOD	<LOD	1.0	101.6 ± 6.7	6.7	100.2 ± 7.0	7.3
								10.0	102.13 ± 2.7	2.7	101.3 ± 3.2	3.2
								20.0	102.1 ± 5.5	5.4	100.9 ± 6.1	6.0
RE	0.1–20	0.9978	0.43	1.41	0.32	<LOD	<LOD	1.0	102.2 ± 5.0	4.9	100.4 ± 5.6	5.6
								10.0	99.4 ± 4.6	4.6	98.7 ± 4.8	4.9
								20.0	100.5 ± 2.8	2.8	99.4 ± 3.5	3.5
BQ	0.1–20	0.9996	0.19	0.61	0.15	<LOD	<LOD	1.0	98.2 ± 10.5	10.8	97.2 ± 11.2	11.5
								10.0	101 ± 2.8	2.8	99.5 ± 4.1	4.1
								20.0	96.5 ± 5.6	5.8	95.3 ± 6.3	6.6

a *n* = 3: number of replicates used for intra-day precision.

b *n* = 9: number of replicates used for inter-day precision over three days.

c LOD = limit of detection; LOQ = limit of quantification; MDL = method detection limit; <LOD = below detection limit.

### 2.6 Ecological risk assessment

Exposure of aquatic organisms to the occurrence of the studied analytes was assessed using the risk quotient (RQ_E_) method, which is the ratio of the measured environmental concentration (MEC) of each analyte in the groundwater sample (mg L^−1^) to the predicted no-effect concentrations (PNEC) (mg L^−1^) values of aquatic organisms (algae, invertebrates, fish) derived from published toxicity data. The PNEC was derived by dividing the available acute median effect/lethal concentration EC_50_ or LC_50_ (mg L^−1^) or chronic (no observed effect concentration (NOEC)) toxicity values (mg L^−1^), as shown in Table 2, by their corresponding assessment factor (AF) of 100 for acute toxicity and 10 for chronic toxicity. This is in accordance with standard regulatory guidance.^35^ Although a formal sensitivity analysis was not performed, these AF values are widely accepted for compounds with limited toxicity data, ensuring a conservative and protective ecological risk assessment. The RQ_E_ levels were classified into three groups according to the RQ values. RQ > 1 indicates a high ecological risk, RQ values between 0.1 < RQ < 1 mean a median risk, and RQ < 0.1 suggests a minimal environmental risk. This classification allows for the identification of phenolic compounds that pose significant threats to aquatic life and informs specific mitigation strategies. The following mathematical equations were used to evaluate risk quotients for the studied PCs in groundwater samples:1
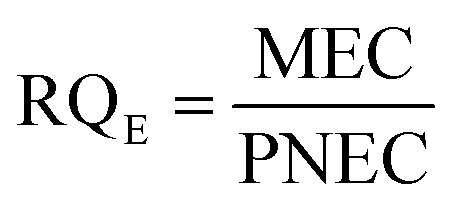
2
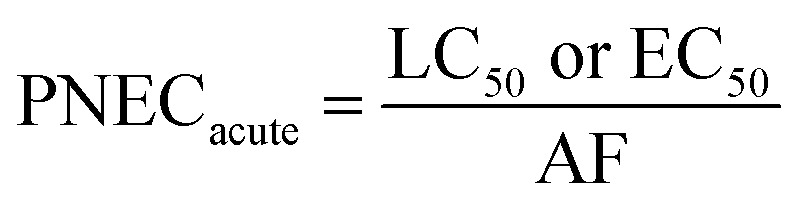
3
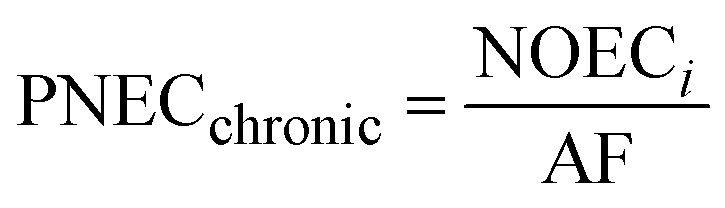


**Table 2:** Acute and chronic toxicity values

Analyte	Trophic level	Toxicity	Toxicity (mg L^−1^)	Ref.
**Acute toxicity**				
BPA	Algae	EC_50_	2.5	36
	Invertebrate	EC_50_	10	
	Fish	LC_50_	4.6	
HQ	Algae	LC_50_	1–4	37
	Invertebrate	LC_50_	0.33	
	Fish	LC_50_	0.638	
BQ	Algae	EC_50_	1.5	37
	Invertebrate	EC_50_	0.059	
	Fish	LC_50_	0.638	
RE	Algae	LC_50_	97	38
	Invertebrate	LC_50_	1.0	
	Fish	LC_50_	53.4	

**Chronic toxicity**				
BPA	Algae	NOEC	1.17	36
	Invertebrate	NOEC	4.11	
	Fish	NOEC	3.64	
HQ	Algae	NOEC	0.019	37
	Invertebrate	NOEC	0.057	
	Fish	NOEC	≥0.066	
BQ	Algae	NOEC	0.019	37
	Invertebrate	NOEC	0.0057	
	Fish	NOEC	≥0.066	
RE	Algae	NOEC	—	38
	Invertebrate	NOEC	0.172	
	Fish	NOEC	≥1.1	

### 2.7 Human health risk assessment

To estimate the human health risk assessment associated with potential non-carcinogenic exposure to the targeted PCs in groundwater, ingestion was identified as the primary exposure pathway due to the significant volume of water typically consumed. The assessment was conducted for both children and adults, using the mean concentrations of the detected compounds in groundwater. This approach reflects realistic exposure scenarios and provides insight into age-specific vulnerability to these contaminants, as evident in previous studies.^12,14,39^ Human exposure to the studied PCs was assessed using the estimated daily intake (EDI), with data obtained from the U.S. EPA 2011 exposure handbook.^40^ Non-carcinogenic risk to human health was computed using the Risk Quotient for Human Health (RQ_H_), derived from the ratio of the EDI to the Drinking Water Equivalent Level (DWEL). The mathematical expressions used in estimating human health risk are expressed in eqn (4)–(6).4
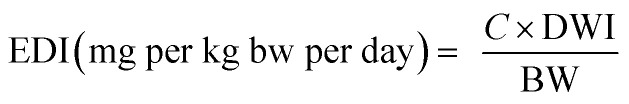
5
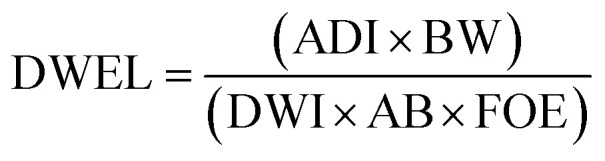
6
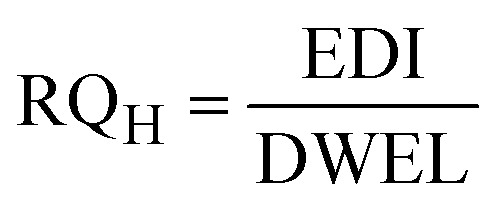
where EDI is the estimated daily intake of the targeted analytes; RQ_H_ represents the non-carcinogenic health risk quotient; and DWEL (Drinking Water Equivalent Level) represents the maximum concentration of a contaminant in drinking water that is not expected to pose any adverse non-carcinogenic health risk to humans over a lifetime of exposure. It is age-specific, accounting for differences in body weight and water intake. RQ_H_ < 0.2 indicates no human health risk, 0.2 ≤ RQH < 1.0 suggests a potential risk to humans, and RQH ≥ 1.0 indicates a potential health risk to humans. Due to the unavailability of standardized reference oral dose (RfD), total daily intake (TDI), or acceptable daily dose (ADI) values for BQ and RE, this study obtained provisional benchmarks from peer-reviewed sources to estimate human health risk assessment. In the absence of an officially established reference dose for BQ, a provisional value of 0.097 mg per kg bw per day was adopted. Although this value is not peer-reviewed, it was applied as a conservative estimate for preliminary risk assessment and is consistent with approaches that benchmark structurally similar compounds.^41^Table 3 presents the parameters used in human health risk assessment for phenolic compounds in groundwater near dumpsites.

**Table 3:** Parameters for evaluation of human health risk assessment of PCs in groundwater

Parameters	Symbol	Unit	Value (adult/children)	Ref.
Concentration	C	mg L^−1^	—	—
Daily water intake	DWI	L d^−1^	2/1	40
Body weight	BW	kg	70/15	42 and 43
Gastrointestinal absorption rate	AB	—	1	4
Frequency of exposure	FOE	—	350/365 = 0.96	4
Acceptable daily dose for BPA	ADI	mg per kg bw per day	0.05	44
Acceptable daily dose for HQ	ADI	mg per kg bw per day	0.04	39
Total daily intake for RE	TDI	mg per kg bw per day	0.04	45
Reference oral dose for BQ	RfD	mg per kg bw per day	0.097	—

### 2.8 Data analysis and interpretation of results

Data analysis was conducted to identify patterns, relationships, and potential risk factors associated with the presence of phenolic compounds in groundwater near dumpsites. Descriptive statistics were used to summarize the concentration ranges, central tendencies, and variability of the analytes. Two-way analysis of variance (ANOVA) was used to assess significant differences in contaminant concentrations across sampling locations and seasons. Pearson correlation analysis was conducted to evaluate linear relationships between phenolic compounds and physicochemical parameters. Principal component analysis (PCA) was used to reduce the dimensionality of the multivariate dataset, simplify interpretation, and identify the dominant sources of variation in phenolic contaminant concentrations and associated physicochemical parameters across groundwater samples. The adequacy of the data for PCA was verified using the Kaiser–Meyer–Olkin (KMO) test and Bartlett's test of sphericity. Components with eigenvalues greater than 1.0 were retained, and factor loadings of ±0.5 or higher were considered significant. Varimax rotation was applied to enhance the interpretability of the extracted components. All statistical analyses were conducted using Excel, GraphPad Prism, and IBM SPSS Statistics version 22, and significance was set at *p* < 0.05.

## 3 Results and discussions

### 3.1 Detection frequency of phenolic compounds

Distinct spatial and seasonal variations in the detection frequency of the targeted PCs were observed, as illustrated in Fig. S1. BPA consistently recorded the highest detection frequency in all three states, with a 100% detection frequency in Osun during the rainy season and similarly high detection rates in Oyo and Lagos States during the dry seasons. In the rural rainy season, BPA was detected in 100% of samples in Osun, followed by 80% in Lagos and 60% in Oyo. During the rural dry season, its frequency remained high in all states, 100% in Oyo, 90% in Osun, and 80% in Lagos. This pattern suggests the widespread presence of BPA irrespective of seasonal differences and highlights its environmental persistence across the sampled regions. In urban areas, BPA was detected at high levels in both seasons, reaching 100% in Lagos during the dry season and 90% in Osun and Oyo across both seasons. However, detection dropped to 50% in Lagos during the urban rainy season. These high detection frequencies and values underscore the broad environmental persistence of BPA and align with the observation that groundwater in proximity to poorly contained dumpsites can receive direct leachate inputs.^46,47^

The detection-frequency patterns for HQ reveal a strong and widespread presence across all study locations, with Osun showing the most pronounced levels. HQ was detected in approximately 80% of rural rainy samples, 90% of rural dry samples, and reached 100% detection in urban rainy areas, highlighting Osun as a hotspot of HQ contamination. Oyo and Lagos also displayed moderate detection frequencies, though less consistently than Osun, suggesting varying intensities of HQ inputs across states. The consistently high detection in both rainy and dry seasons indicates that HQ contamination is persistent and not strongly driven by seasonal changes. Instead, the pattern suggests a steady, continuous release of HQ likely from domestic, cosmetic, and small-scale industrial activities, resulting in its frequent occurrence across diverse hydrogeological settings. Literature confirms its persistent presence due to widespread use and improper disposal, rather than sporadic pollution.^39^ RE exhibited moderate detection frequencies ranging from 50–70%. In the rural rainy season, detection was moderate in Oyo (50%) and lower in Osun and Lagos (40%). In the rural dry season, Osun and Lagos showed significant increases (90% and 80% respectively), while Oyo remained unchanged at 50%. Urban detection of RE surged in Lagos during the dry season (100%) but was lower during the rainy season (50%), whereas Osun and Oyo showed moderate levels (30–70%) across both seasons. BQ, generally the least detected compound, demonstrated stark seasonal and spatial variation. In rural rainy conditions, BQ detection peaked in Lagos, with lower rates in Oyo (30%) and Osun (20%). However, rural dry season levels spiked dramatically in Osun (90%) but dropped to 30% in Oyo and 0% in Lagos. In urban settings, BQ was highly prevalent in Oyo and Lagos during the rainy (80% and 60%) and dry (50% and 90%) seasons, while Osun maintained lower frequencies, though it rose from 10% in the rainy to 60% in the dry season.

Across the three states, Osun consistently recorded high detection frequencies for all compounds, particularly BPA and HQ, across both rural and urban settings. Lagos exhibited the highest urban dry season frequencies, especially for BPA, RE, and BQ, likely reflecting intensified anthropogenic activities such as industrial effluent discharge. Oyo generally showed lower detection rates for HQ and RE, especially in urban dry periods, pointing to regional differences in pollution sources, land use, and environmental management practices.

### 3.2 Spatiotemporal distribution of phenolic compounds

**Fig. 2:**
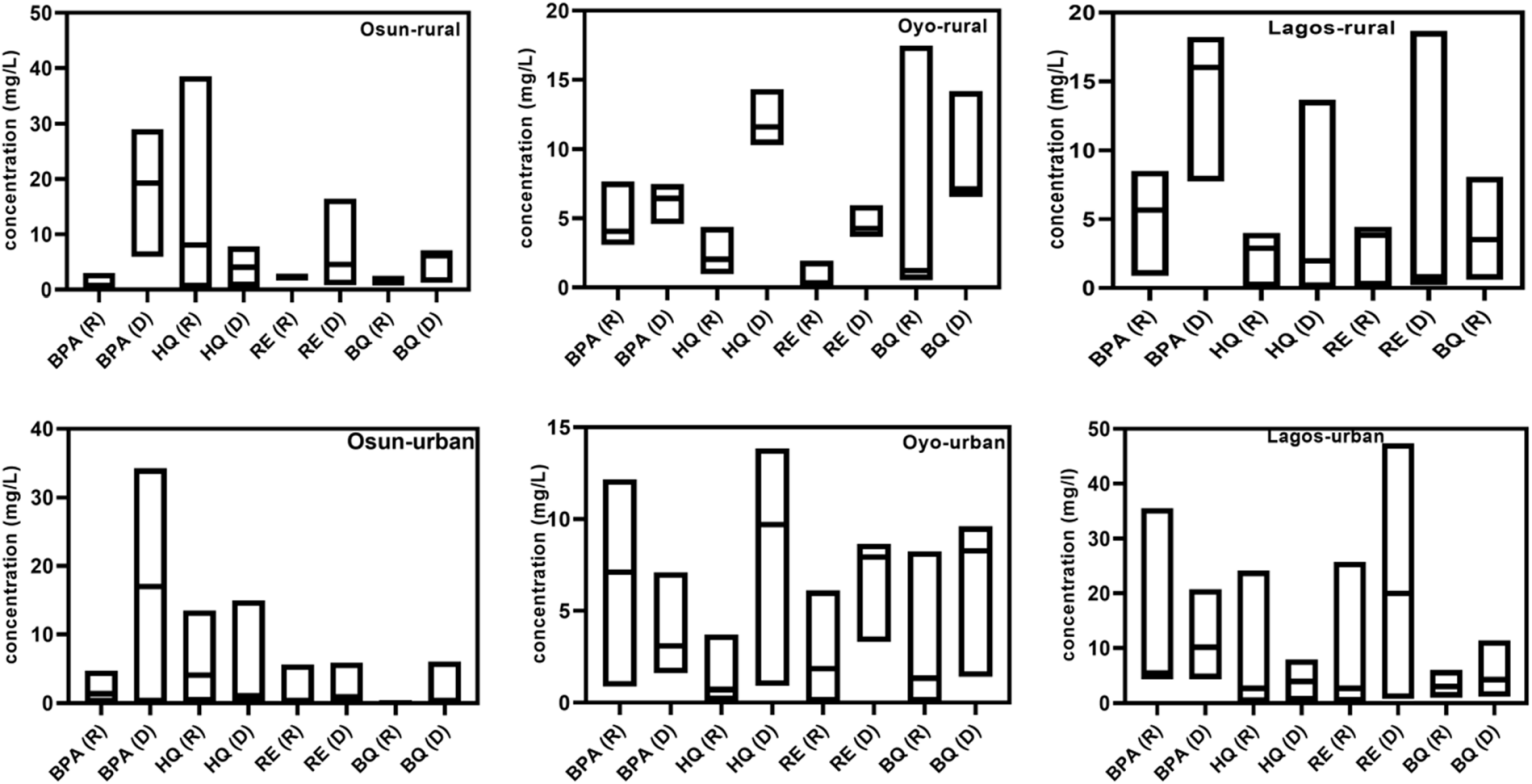
Box plot concentration of Bisphenol A (BPA), Hydroquinone (HQ), Resorcinol (RE), and Benzoquinone (BQ) in urban and rural sites of Osun, Oyo, and Lagos States groundwater samples during the rainy (R) and dry (D) seasons (*n* = 60 per season).

The spatiotemporal distribution and statistical summaries of BPA, HQ, RE, and BQ in groundwater across Osun, Oyo, and Lagos States for both rainy and dry seasons are presented in Tables SI 3, 4 and Fig. 2. The result revealed distinct seasonal and geographical patterns influenced by proximity to dumpsites and land-use characteristics. Across most locations, dry-season concentrations were higher than rainy-season values, a trend especially pronounced for BPA and RE, indicative of accumulation during periods of reduced water table and limited flushing.

BPA showed the clearest seasonal contrast, with consistently higher dry-season levels in both rural and urban groundwater. In rural Osun, mean BPA concentrations increased from 1.06 ± 0.90 mg L^−1^ (rainy) to 19.16 ± 6.47 mg L^−1^ (dry), with medians rising from 0.79 to 19.29 mg L^−1^ (Table SI 4). Similar dry-season elevations were noted in rural Lagos (4.50 ± 2.84 to 14.42 ± 4.18 mg L^−1^) and Oyo (4.82 ± 2.04 to 6.36 ± 1.00 mg L^−1^). Urban groundwater generally mirrored this trend, with Osun (1.88 ± 1.38 to 20.90 ± 9.54 mg L^−1^) and Lagos (11.10 ± 13.69 to 11.90 ± 5.67 mg L^−1^) showing elevated dry-season concentrations. The exception was Oyo urban sites, where rainy-season levels (0.85–12.2 mg L^−1^; median 7.11 mg L^−1^) exceeded dry-season values (1.60–7.11 mg L; median 3.08 mg L^−1^), likely due to rainfall-driven mobilization of surface contaminants into the shallow aquifers. High variability in Lagos urban BPA during the rainy season (mean 11.10 ± 13.69 mg L^−1^) may reflect episodic leachate inflows from poorly managed waste or localized industrial discharge. BPA's hydrophobicity and semi-volatility likely favour its persistence under dry-season conditions.

HQ demonstrated substantial variability, consistent with its high solubility and redox reactivity. In rural groundwater, concentrations ranged from ND–18.20 mg L^−1^ (rainy) and ND–7.80 mg L^−1^ (dry) in Osun; ND–4.37 mg L^−1^ (rainy) and ND–14.32 mg L^−1^ (dry) in Oyo; and ND–3.21 mg L^−1^ (rainy) to ND–13.68 mg L^−1^ (dry) in Lagos. Urban groundwater recorded higher magnitudes, especially in Lagos, where rainy-season levels reached 0.06–24.13 mg L^−1^. HQ is highly water-soluble and redox-active, making it susceptible to both microbial transformation and mobility in aqueous environments. Its higher rainy season presence in Lagos may be attributed to increased runoff mobilizing surface contaminants into aquifers, while its dry season persistence in Oyo may reflect deeper infiltration from surrounding waste leachate.

RE was detected at moderate levels but displayed clear seasonal differences. Rural groundwater showed increases from ND–2.87 mg L^−1^ (rainy) to 0.79–16.43 mg L^−1^ (dry) in Osun, and similar dry-season elevations in Oyo and Lagos. Urban samples followed this trend, with Lagos recording particularly elevated dry-season concentrations, reaching 47.37 mg L^−1^. As a dihydroxybenzene isomer with moderate solubility and a tendency to persist under anaerobic conditions, RE's dry-season prevalence may be linked to reduced oxygen availability near dumpsites and slower biodegradation rates.

BQ exhibited the most irregular detection pattern among the studied PCs. Rural concentrations ranged from 0.74–2.48 mg L^−1^ (rainy) and 1.23–7.14 mg L^−1^ (dry) in Osun; 0.54–17.48 mg L^−1^ (rainy) and 6.55–14.17 mg L^−1^ (dry) in Oyo; whereas Lagos showed BQ only during the rainy season, consistent with its low detection frequency. Urban samples were similarly variable, with values generally spanning ND to low mg L^−1^ levels across sites and seasons. As a redox-active degradation product of HQ, BQ's presence may be influenced by microbial activity, oxidative conditions, and pH fluctuations. Its sporadic detection may reflect a transient transformation from precursors or rapid environmental degradation under sunlight or aerobic conditions.

Overall, groundwater near dumpsites and in urban areas showed higher contamination levels for the studied PCs, consistent with the mean and median values reported in Tables SI 3 and 4. The large standard deviations, particularly in urban Lagos and Osun, indicate substantial spatiotemporal heterogeneity, likely influenced by sporadic leachate inflows, localized point-source discharges, and site-specific factors such as soil type, groundwater depth, and hydrological connectivity. It further shows that the contaminants vary widely across sampling points, affirming the importance of considering both central tendency and variability when assessing groundwater contamination and potential risks.

### 3.3 Spatial and seasonal influences on PCs concentrations across states

Given that groundwater samples were collected in proximity to open dumpsites, substantial variability in contaminant levels was expected due to differential leaching, waste composition, and hydrological conditions. To robustly assess the influence of both spatial (urban *vs.* rural) and seasonal (rainy *vs.* dry) factors, a two-way ANOVA with replication was performed, and the result is presented in Table SI 5. This statistical approach is appropriate for replicated environmental datasets and enables the detection of main and interaction effects, accounting for the inherent variability present in field-based sampling near pollution hotspots. The results revealed notable differences in pollution dynamics between the states.

In Osun, both the effect of location (*F* = 6.50, *p* = 0.012) and compound–season variation (*F* = 4.48, *p* < 0.001) were statistically significant, while the interaction effect was not. This suggests that compound concentrations differ significantly between rural and urban sites and across seasons, but seasonal trends were largely consistent across both spatial contexts. The strong seasonal effect may be attributed to reduced dilution and pronounced concentration effects during the dry season, driven by lower water tables and decreased groundwater recharge, which collectively lead to higher apparent contaminant levels. Conversely, the spatial variation likely reflects differences in human, agricultural activities and local hydrogeological settings across the studied sites.

Oyo samples showed a significant effect of compound–season combinations only (*F* = 3.80, *p* = 0.0008), while location (*F* = 0.34, *p* = 0.563) and interaction (*F* = 1.76, *p* = 0.101) effects were not significant. This suggests that while seasonal fluctuations in compound concentrations exist, the spatial distribution of contaminants between rural and urban areas is relatively uniform. This could indicate more diffuse sources of pollution or more homogeneous land use practices and environmental conditions across the sampling areas in Oyo.

For Lagos, all three factors were statistically significant: location (*F* = 12.82, *p* = 0.0005), compound–season combinations (*F* = 9.39, *p* < 0.000001), and interaction (*F* = 4.48, *p* = 0.0002). These findings indicate that both spatial and seasonal variations strongly influenced contaminant levels and that these effects were not uniform across sampling areas. The significant interaction effect suggests localized seasonal dynamics, possibly driven by intensified industrial discharge, urban runoff, and higher population density. Higher dry-season concentrations in urban locations may also reflect reduced dilution, increased surface runoff accumulation, or leachate infiltration from surrounding dumpsites.

Generally, the observed differences across states are likely driven by regional differences in land use intensity, population density, industrial activity, and environmental management practices. Lagos, as Nigeria's most urbanized and industrialized state, displayed the most pronounced anthropogenic influence on groundwater quality. Osun, with a mixed urban–rural profile and substantial agricultural activity, showed significant but more consistent variation, while Oyo demonstrated relatively uniform pollution trends, possibly due to more balanced rural–urban development and less concentrated industrial zones.

### 3.4 Global comparison of the studied PCs in groundwater near dumpsites

BPA is widely used in the plastic industry as a non-polymeric additive, with further applications in automobile components, electrical and electronic equipment, and in the production of thermal paper.^48^ In this study, BPA exhibited high detection frequencies across all states and seasons, consistently exceeding 80% in most locations. This widespread occurrence reflects its environmental persistence and extensive use, as well as its mobility in soil and groundwater. While there is a paucity of studies specifically examining BPA contamination in groundwater near open municipal waste dumpsites in Sub-Saharan Africa, existing literature suggests that BPA contamination of water sources near landfills is common in other parts of the world. For instance, Zhang *et al.* (2019) reported the presence of BPA in drinking water treatment plants. Noticeable regional differences were observed in the concentration and distribution patterns, with BPA being the most predominant in most source water and drinking water samples. However, the estimated daily intake (EDI) values suggested relatively low exposure levels for the general population.^49^ Another study also showed the persistence of BPA in drinking water, with a relatively higher detection frequency during the dry season, although the human health effects were deemed negligible. The elevated concentration of BPA detected during the dry season seems to agree with the findings of this study.^50^ Recent studies have also reported a widespread occurrence of BPA (0–0.00016 mg L^−1^) in groundwater samples close to municipal landfills.^51,52^ It is worth noting that BPA groundwater contamination, caused by leachate infiltration, has also been scarcely reported globally. However, research on BPA contamination in rivers, well water, soil, and sediments has been documented.^53^ For instance, Yamamoto *et al.* (2001) reported BPA concentrations in landfill leachates ranging from 1.3 to 17.2 mg L^−1^.^54^ Similarly, Masoner *et al.* (2014) revealed high detection frequency in leachate samples across the United States, revealing that municipal waste sites can act as major source points capable of releasing PCs into groundwater aquifers.^55^ The high mean BPA levels (Table SI 3) reflect its widespread aquifer contamination, driven by extensive single-use of plastic ware, poor disposal practices, and its strong leaching potential and persistence under Nigeria's tropical conditions.^56^ In subsurface environments, particularly under anaerobic conditions, BPA degradation is reduced or nearly absent. This is due to the limited availability of oxygen, which suppresses oxidative metabolic pathways essential for microbial breakdown, thereby enhancing BPA's long-term stability and persistence in groundwater systems.^31,57^

The hydroquinone concentrations observed in this study underscore its environmental persistence, especially under subsurface conditions. Comparative groundwater data for HQ, particularly in sub-Saharan Africa, remains extremely scarce. The value reported in Table SI 6 was obtained from published supplementary data associated with in the first African study to quantify HQ in groundwater, reported by Otitoju *et al.* (2023).^12^ In this study, HQ concentrations peaked up to 18.2 mg L^−1^ and were several thousand-fold higher than the previous study. These elevated concentrations strongly indicate significant leachate infiltration from nearby open dumpsites, intensified by shallow, unconfined aquifers and highly permeable subsurface soils that facilitate rapid contaminant migration and accumulation. Similarly, global data on BQ and RE in groundwater remain scarce, limiting comparative assessment. Nevertheless, the concentrations of BQ and RE reported in this study raise substantial environmental and public health concerns, underscoring the need for further investigation and monitoring. Furthermore, both HQ and BQ pose a higher ecological risk in groundwater even at low concentrations due to their acute and chronic toxicity. Although RE is less toxic, it may present risks in heavily contaminated environments. The persistence of these PCs under anaerobic groundwater conditions may intensify their potential risk to both aquatic life and human health, especially in areas near open dumpsites or unlined landfills.

Overall, this study confirmed the widespread occurrence of the four targeted PCs in groundwater samples from both rural and urban areas across Osun, Oyo, and Lagos States during the rainy and dry seasons. This is quite expected as the majority of the studied sites lack well-engineered dumpsites, leading to unrestricted percolation of leachate into shallow aquifers. The underlying aquifers are predominantly unconfined, highly fractured, and characterized by sandy or lateritic soils with high hydraulic conductivity and conditions.^58^ These conditions facilitate rapid contaminant transport with limited dilution. Additionally, the dumpsite receives mixed municipal, industrial, and electronic waste without segregation, which is known to increase the release of PCs and other organic contaminants to levels higher than those reported in more developed climes.^59–61^ Moreover, the age of the sampled dumpsites ranged from 10 to 30 years and contributed significantly to the elevated concentrations detected in this study.^62^ These combined factors provide a plausible explanation for the high detection frequencies and support the observed magnitudes of contamination reported in this study.^63^ The result also revealed notable spatiotemporal variations with generally higher concentrations and detection frequencies observed during the dry season, indicating possible seasonal accumulation due to reduced dilution, increased contaminant leaching, and loading. Concentrations below LOD values were reported as non-detected (ND) and treated as zero for all statistical summaries and exposure calculations. This approach was adopted to prevent any misrepresentation of mean concentrations and pollution burden to both human and aquatic organisms. Comparative global data on the targeted PCs in groundwater based on available literature are summarized in Table SI 6.

### 3.5 Ecological risk assessment

**Fig. 3:**
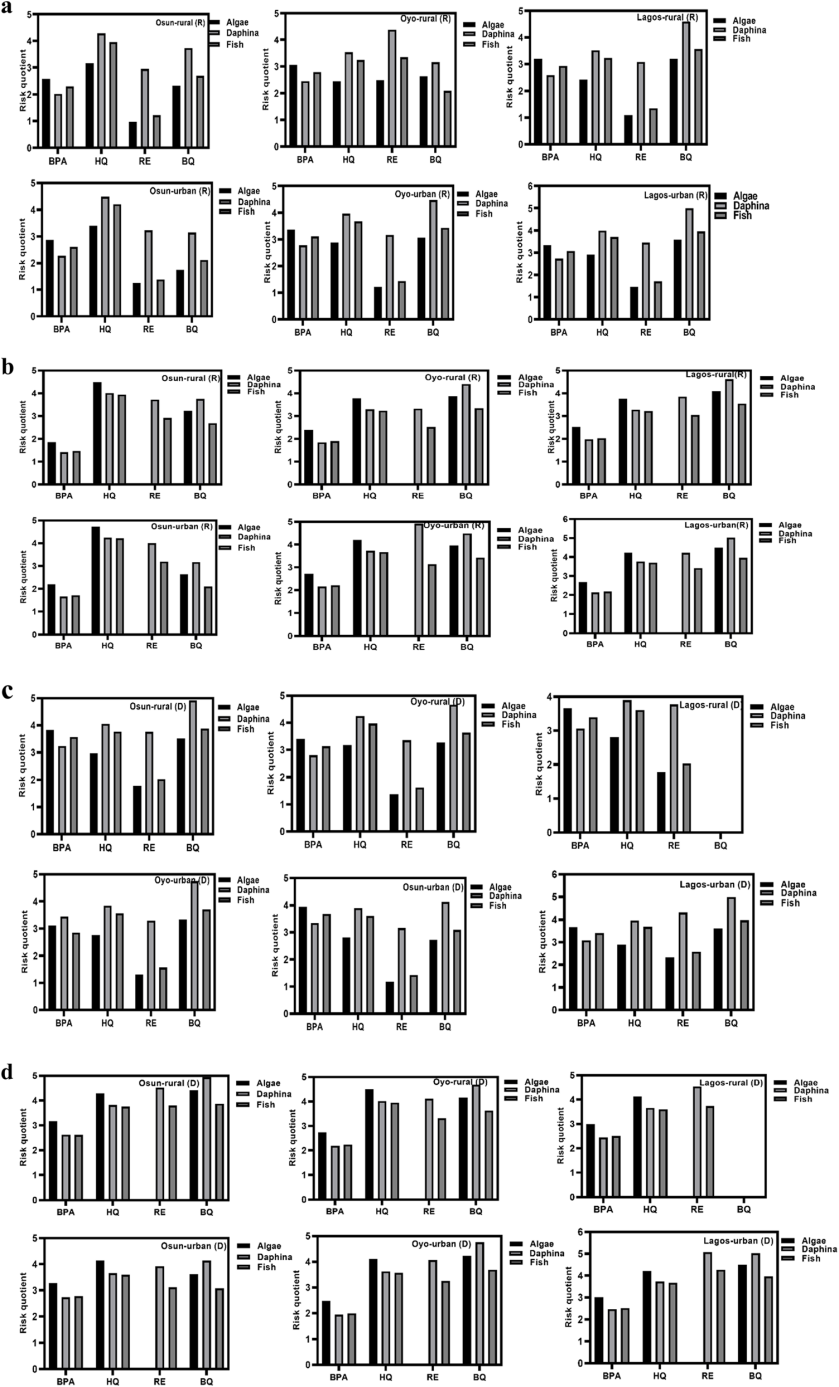
(a) Acute RQ_E_ for Bisphenol A (BPA), Hydroquinone (HQ), Resorcinol (RE), and Benzoquinone (BQ) during the rainy season (R) in rural and urban areas of Osun, Oyo, and Lagos States (*n* = 60 per season). (b) Chronic RQ_E_ for Bisphenol A (BPA), Hydroquinone (HQ), Resorcinol (RE), and Benzoquinone (BQ) during the rainy season (R) in rural and urban areas of Osun, Oyo, and Lagos States (*n* = 60 per season). (c) Acute RQ_E_ for Bisphenol A (BPA), Hydroquinone (HQ), Resorcinol (RE), and Benzoquinone (BQ) during the dry season (D) in rural and urban areas of Osun, Oyo, and Lagos States (*n* = 60 per season). (d) Chronic RQ_E_ for Bisphenol A (BPA), Hydroquinone (HQ), Resorcinol (RE), and Benzoquinone (BQ) during the dry season (D) in rural and urban areas of Osun, Oyo, and Lagos States (*n* = 60 per season).

In this study, ecological risk assessment was evaluated for the four PCs in groundwater samples using the available acute and chronic data from the literature for algae, daphnia, and fish. RQ_E_ values were benchmarked against U.S. EPA ecological Levels of Concern (LOCs), 0.5 for acute effects and 1.0 for chronic effects.^64^ While HQ chronic toxicity data obtained from the Australian Government Department of Health and Aged Care (Australian Industrial Chemicals Introduction Scheme)-Hydroquinone and *p*-benzoquinone Evaluation Statement (2022) was used in estimating the chronic ecological risk associated with BQ in this study. Seasonal and spatial assessments are depicted in Fig. 3a–d.

In rural Osun groundwater, HQ had the highest RQ_E_ values (4.1–4.9), with daphnia being at a more intense ecological risk. However, the RQ_E_ values were higher in urban environments, with other taxonomic groups being equally at great risk. BQ showed elevated RQ_E_, particularly in rural settings, while BPA, the parent compound, was equally above the required threshold across both settings. Although RE displayed the lowest RQ_E_ values among the analyzed compounds, it still surpassed the RQ_E_ > 1 benchmark, signaling meaningful ecological concern.

Oyo rural groundwater samples posed a serious ecological risk to the aquatic organisms, with all four PCs exceeding the acute and chronic threshold of 1 across all taxa. Daphnia emerged as the most vulnerable, followed by algae and fish. In Lagos, the urban groundwater recorded higher RQ_E_ values, with BQ being conspicuously high and significantly threatening to aquatic organisms. Both BPA and HQ were comparatively high, while RE remained the lowest. Nonetheless, the four PCs were high across both settings, and the acute RQ for the taxonomic group followed this trend: daphnia > algae > fish. The chronic ecotoxicity value for RE in algae was not available in the literature and, accordingly, was omitted from the chronic risk assessment for this taxonomy.

Notably, the chronic RQ_ES_ for RE and BQ were substantially higher than those for the parent compound BPA, suggesting that oxidative degradation of BPA in the enclosed aquatic environment may have produced more persistent or toxic byproducts. It should be noted that the derivation of the PNEC and provisional RfD for BQ involves uncertainties due to limited toxicological data. Nevertheless, the consistently high concentrations observed in this study indicate a potential ecological concern, suggesting that even conservative assumptions would likely identify elevated risk and necessitate precautionary mitigation measures.

Alarmingly, 96.03% of the chronic RQ_E_ values calculated in this study exceeded the ecological benchmark of 1.0, indicating that immediate remediation strategies are necessary. During the dry season, higher acute and chronic RQ_E_ values were recorded for the PCs, underscoring the impact of seasonal variation, reduced dilution capacity, and increased contamination loads. This pattern aligns with published findings showing elevated ecological risk during dry periods due to lower water volumes and reduced hydrodynamic flow.^12,65^ Generally, 98.03% of the sampled sites exhibited RQ_E_ values exceeding the ecological threshold (RQ_E_ > 1), posing serious ecological concerns. The persistently high concentrations detected in this study indicate that urgent mitigation approaches are imperative to protect the aquatic ecosystem in this region.

### 3.6 Human health risk assessment

**Fig. 4:**
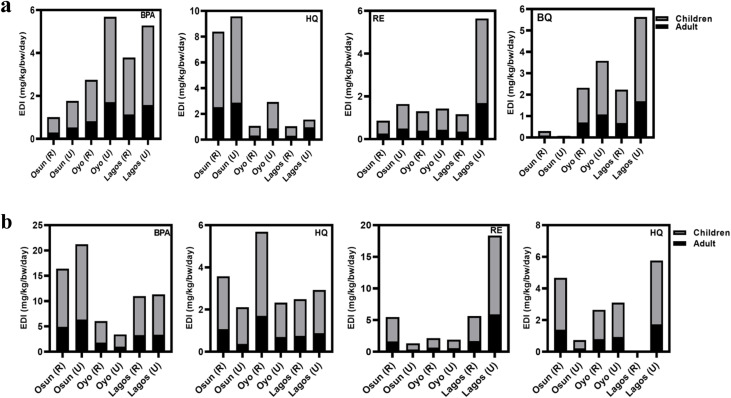
(a) Estimated daily intake for Bisphenol A (BPA), Hydroquinone (HQ), Resorcinol (RE), and Benzoquinone (BQ) in rural (R) and urban (U) groundwater samples of Osun, Oyo, and Lagos during the rainy season (*n* = 60 per season). (b) Estimated Daily Intake Bisphenol A (BPA), Hydroquinone (HQ), Resorcinol (RE), and Benzoquinone (BQ) in rural (R) and urban (U) groundwater samples of Osun, Oyo, and Lagos during the dry season (*n* = 60 per season).

**Fig. 5:**
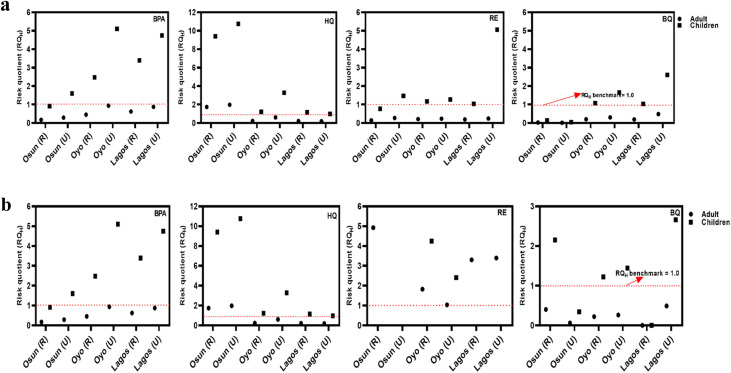
(a) Human health risk quotient for Bisphenol A (BPA), Hydroquinone (HQ), Resorcinol (RE), and Benzoquinone (BQ) in rural (R) and urban (U) groundwater samples of Osun, Oyo, and Lagos during the rainy season (*n* = 60 per season). (b) Human health risk quotient for Bisphenol A (BPA), Hydroquinone (HQ), Resorcinol (RE), and Benzoquinone (BQ) in rural (R) and urban (U) groundwater samples of Osun, Oyo, and Lagos during the dry season (*n* = 60 per season).

Groundwater remains a predominant source of drinking water supply in Nigeria, especially in the underserved areas. Therefore, the estimation of human health exposure to these USEPA water priority pollutants (HQ, BQ, and RE) and the contaminant of emerging concern (BPA) must be evaluated. Human health exposure and non-cancer risk were evaluated for two main groups (children and adults) based on the USEPA 2011 exposure handbook using the mean concentrations of PCs obtained in this study. Findings from computing EDI and RQ_H_ are presented in Fig. 4a and b, as well as Fig. 5a and b.

Currently, there are no established permissible concentration limits for RE and BQ by either the United States Environmental Protection Agency (USEPA) or the World Health Organization (WHO). However, the USEPA has reported a no-observed-adverse-effect level (NOAEL) of 4.3 mg per kg bw per day for HQ in drinking water, based on chronic human exposure studies. The European Union Directive (EU) 2020/2184 has also set a parametric value of 0.0025 mg L^−1^ for BPA in drinking water, which will take effect on 12 January 2026.

Across all three states and both seasons (Fig. 4a and b), 90% of EDI exceeded the established limit, indicating potential serious health risks through endocrine disruption and oxidative stress.^66^ Seasonal differences were evident as the EDI values for BPA, RE, and BQ were significantly higher during the dry season, whereas HQ exhibited higher EDI values in the rainy season. The higher EDI values for BPA, RE, and BQ during the dry season are likely due to their moderate hydrophobicity and lower water solubility, which promote accumulation in groundwater under reduced dilution and higher temperatures that enhance desorption from contaminated surfaces. HQ, being more hydrophilic and highly water-soluble, showed higher EDI values during the rainy season, likely due to increased runoff, enhanced leaching from open dumpsites, and the formation of HQ as a degradation product of other PCs under elevated microbial activity.

As shown in Fig. 5a and b, non-cancer health risk assessment based on RQ_H_ values revealed that children face a higher risk than adults, largely due to their elevated intake-to-body weight ratios. RQ_H_ values for BPA and HQ exceeded the safety threshold of 1, while BQ displayed significantly higher RQ_H_ values, reaching 11.2. This elevated risk is influenced by the physicochemical properties of these compounds. BPA and HQ, being moderately hydrophilic and persistent, readily leach into groundwater from plastic waste and the oxidative degradation of phenolic precursors. BQ, a reactive electrophilic compound formed from the oxidation of hydroquinone or aromatic hydrocarbons, exhibits high mobility and acute toxicity, especially under oxidizing conditions often found near unmanaged waste sites.

As expected, samples from urban locations consistently showed higher RQ_H_ values than those from rural areas, likely due to more intense anthropogenic pressures, including industrial effluents, higher population density, and increased leaching and infiltration from open dumpsites. Notably, BQ and RE values increased during the rainy season, potentially due to oxidative transformation of precursors such as HQ and RE, as well as surface runoff that facilitates contaminant mobilization into aquifers. Despite seasonal variation, 40% of all samples exceeded the non-cancer health risk threshold, indicating widespread exposure. Generally, both EDI and RQ_H_ values were higher during the dry season, potentially due to lower dilution, longer pollutant residence times, and higher concentrations resulting from leachate accumulation.

### 3.7 Principal component analysis (PCA)

Principal component analysis (PCA) was conducted on groundwater samples from Osun, Oyo, and Lagos States during both the rainy and dry seasons to assess spatial and seasonal trends in phenolic contamination. Mean environmental concentrations were first transformed and normalized using Kaiser's normalization rule, followed by Varimax rotation to simplify factor analysis. The resulting variable loadings are summarized in Table 4. The suitability of the dataset for factor analysis was confirmed using the Kaiser–Meyer–Olkin (KMO) measure of sampling adequacy and Bartlett's test of sphericity. A KMO value ≥0.5 indicates acceptable sampling adequacy, while a significant Bartlett's test (*p* < 0.001) confirms sufficient inter-variable correlations for PCA. In this study, the dataset showed a KMO of 0.78 and a significant Bartlett's test (*p* < 0.001), validating the use of PCA. Compounds with no detection frequency were excluded. Three principal components (PCs) were extracted for each location, each with eigenvalues >1.0, cumulatively explaining 70.17–79.47% of the total variance. The PCA revealed that groundwater phenolic contamination exhibited clear spatial and seasonal variability, reflecting the combined influence of ionic parameters and anthropogenic activities.

**Table 4:** Rotated component matrix for variables in GW sample from Osun, Oyo, and Lagos States^a^

	Osun (R)			Osun (U)			Oyo (R)			Oyo (U)			Lagos (R)			Lagos (U)		
	1	2	3	1	2	3	1	2	3	1	2	3	1	2	3	1	2	3
BPA_R	−0.11	**0.97**	−0.10	−0.19	0.05	−0.05	**−**0.29	0.11	**0.83**	−0.61	**0.66**	−0.36	**0.64**	0.48	0.12	−0.61	0.66	**-0.36**
BPA_D	−0.11	**0.97**	−0.10	−0.16	**0.86**	−0.25	0.27	0.30	**0.59**	**0.65**	−0.40	−0.54	−0.31	0.32	−0.17	**0.65**	−0.40	**0.54**
HQ_R	0.13	**0.86**	0.31	0.19	**0.53**	−0.37	**0.95**	−0.04	0.22	0.15	**0.93**	0.06	**0.91**	0.04	0.17	−0.15	**0.94**	0.06
HQ_D	**0.92**	0.08	−0.21	0.14	−0.79	−0.03	−0.10	**0.88**	−0.06	0.07	0.07	−0.88	0.17	**0.82**	0.13	0.07	**0.07**	−0.88
RE_R	−0.12	0.20	**0.87**	0.23	−0.38	0.42	**0.83**	0.25	−0.76	−0.76	0.21	0.03	0.20	0.25	−0.39	−0.76	0.21	0.03
RE_D	0.16	−0.54	−0.14	0.28	0.09	**0.88**	**0.52**	**0.65**	0.24	0.08	0.08	**0.77**	0.07	−0.91	0.19	0.24	0.08	**0.77**
BQ_R	**0.86**	−0.02	0.04	**0.81**	−0.42	−0.02	**0.95**	0.04	−0.08	−0.06	**0.93**	0.18	**0.91**	−0.18	0.16	−0.06	**0.93**	0.17
BQ_D	−0.07	−0.08	**0.83**	−0.08	−0.15	**0.95**	−0.09	0.17	−0.76	0.34	0.45	**0.68**	0.48	−0.77	−0.18	−0.34	0.45	**0.68**
pH	−0.13	−0.14	0.07	−0.43	**0.54**	−0.08	−0.66	−0.40	−0.18	**0.56**	0.32	−0.35	−0.01	0.13	−0.89	**0.56**	0.32	−0.35
EC	**0.96**	−0.18	−0.05	**0.93**	0.03	0.14	0.38	**0.81**	0.42	**0.82**	−0.08	−0.36	0.18	0.11	**0.91**	**0.82**	−0.08	0.36
TDS	**0.97**	−0.18	−0.05	**0.96**	0.01	−0.01	**0.56**	**0.52**	0.32	**0.82**	−0.06	0.35	0.39	0.11	**0.75**	**0.82**	−0.07	0.35
Eigen values	3.96	3.04	1.59	3.61	2.37	1.75	4.78	2.05	1.52	3.86	3.02	1.74	3.75	2.55	2.45	3.86	3.02	1.74
% Of variance	36.03	27.61	14.46	32.78	21.53	15.87	43.51	18.62	13.85	35.10	27.47	15.78	34.07	23.15	22.25	29.49	24.65	24.22
% cumulative	36.03	63.63	78.09	32.78	54.30	70.17	43.51	62.13	75.96	35.10	62.57	78.35	34.07	57.22	79.47	29.49	54.14	78.36

a N.B.: extraction method: principal component analysis with Varimax rotation and Kaiser normalization. Factor loadings ≥0.5 are highlighted in bold.

In Osun, rural groundwater was primarily influenced by ionic strength and quinone inputs, as PC1 (36.03%) showed strong positive loadings from TDS (0.97), EC (0.96), BQ_R (0.86), and HQ_D (0.92). PC2 (27.61%), dominated by BPA_R/D (0.97) and HQ_R (0.86), indicated plastic-related pollution due to BPA, while HQ potentially originates from co-occurring industrial or pharmaceutical sources. PC3 captured seasonal variability through high RE_R (0.87) and BQ_D (0.83). Similarly, in Osun urban, PC1 (32.80%) mirrored rural trends with dominant EC and TDS contributions, alongside BQ_R (0.81), emphasizing ionic and organic pollutant buildup during rainfall. PC2 (21.53%) highlighted BPA_D (0.86), likely linked to dry-season accumulation, while PC3 (15.90%) reflected seasonal leaching or degradation of resorcinol, as evidenced by strong loadings on RE_D (0.88) and RE_R (0.42).

For Oyo, rural groundwater exhibited distinct quinone-driven contamination, as PC1 (43.51%) was heavily loaded by HQ_R (0.95) and BQ_R (0.95), suggesting redox-sensitive inputs from domestic or agricultural activities. PC2 (18.62%), with high EC (0.81) and RE_D (0.65), indicated seasonal ionic shifts affecting resorcinol, while PC3 (13.85%) captured BPA_R (0.83), possibly from wet-season plastic leachates. In contrast, Oyo urban groundwater was strongly influenced by TDS (0.82), EC (0.82), and BPA_D (0.65) in PC1 (35.10%), reflecting salinity and polymer-related pollution. PC2 (27.47%) emphasized BPA_R (0.66) and HQ_R (0.93), and PC3 (15.78%) reflected broader chemical interactions involving RE_D (0.77) and BQ_D (0.68).

Lagos rural samples showed dominant quinone contamination under wet conditions, with PC1 (34.07%) governed by HQ_R (0.91) and BQ_R (0.91). PC2 (23.15%) revealed strong negative loading for RE_D (−0.91), suggesting *in situ* transformation or microbial degradation, while PC3 (22.25%) linked TDS (0.75) and BQ_D (−0.18), implicating moderate salinity influence. In Lagos Urban, PC1 (29.49%) was shaped by TDS (0.82), EC (0.82), and BQ_R (0.93), underscoring the role of industrial runoff and landfill leachates, particularly during the rainy season. PC2 (24.65%) identified strong seasonal behavior in BPA_R (0.66) and RE_D (−0.77), while PC3 (24.22%) further reinforced this trend with high loadings for BQ_D (0.68) and RE_D (0.77). Collectively, PCA revealed that groundwater phenolic contamination is strongly influenced by ionic strength, seasonal fluctuations, and land-use type. The sampling areas in rural areas exhibited clearer seasonality and source-specific patterns, whereas urban locations displayed greater chemical complexity and multiple pollutant sources, reflecting both anthropogenic pressure and environmental transformations into daughter compounds that may be more toxic than their parent compound.^67^

### 3.8 Pearson correlation coefficient

Fig. S4 explains the comparative Pearson correlation analysis (*r*) across Osun, Oyo, and Lagos States. The resultsshowed pronounced differences between rural and urban groundwater samples in the behaviour, transformation pathways, and geochemical controls of phenolic contaminants in groundwater. Correlation strength was interpreted as strong (|*r*| ≥ 0.70), moderate (0.50 ≤ |*r*| < 0.70), and weak (|*r*| < 0.50). Across most rural sites, particularly in Osun and Oyo, parent phenolic compounds exhibited strong positive correlations with their corresponding degradation products, particularly between hydroquinone and benzoquinone (*r* ≥ 0.70), alongside strong to moderate correlations with pH, electrical conductivity (EC), and total dissolved solids (TDS). These consistent relationships indicate that phenolic transformation in rural aquifers is largely governed by intrinsic hydrogeochemical and redox-mediated processes operating under relatively stable subsurface conditions. Urban groundwater across the three states shows weaker or negative correlations between parent and degraded species, reflecting multiple and continuous anthropogenic inputs that disrupt systematic degradation trends. Although selective compounds, notably resorcinol and benzoquinone, maintained strong relationships with pH and mineralization in urban aquifers, especially in Lagos. Generally, the results across all six sites demonstrated a clear rural-urban gradient in phenolic fate and transport, with rural groundwater chemistry dominated by coherent geochemical controls and urban aquifers characterized by complex, input-driven contamination patterns. These findings underscore the importance of land-use-specific groundwater monitoring, risk assessment, and management strategies for phenolic contaminants across Southwestern Nigeria.

## 4 Conclusion

This study presents the first year-long, different states assessment of BPA, HQ, RE, and BQ in groundwater systems proximate to open dumpsites across Southwestern Nigeria. The findings confirmed widespread contamination with pronounced seasonal and spatial variations across Osun, Oyo, and Lagos States. BPA consistently exhibited the highest concentrations, reaching up to 20.90 mg L^−1^ in urban Osun during the dry season, while RE and BQ peaked notably in urban Lagos and rural Oyo, respectively. HQ displayed variable but significant elevations in both Osun and Oyo. Seasonal patterns, particularly dry-season accumulation, were driven by reduced hydrological dilution, shallow unconfined aquifers, permeable sandy–lateritic soils, and direct leachate infiltration from unlined dumpsites. Continuous anthropogenic inputs, including domestic waste and extensive use of plastics, further contributed to elevated contaminant levels. Seasonal redox fluctuations likely influenced the transformation and mobility of HQ and BQ, highlighting the dynamic nature of phenolic compound behaviour in these groundwater systems. Risk assessments indicated potential ecological and human health concerns. Hazard quotients for sensitive aquatic taxa exceeded stipulated limits, while estimated daily intakes for children often surpassed reference doses, signaling significant non-carcinogenic risks. Principal component analysis elucidated clear seasonal and spatial trends in groundwater contamination, with EC, TDS, and quinones (HQ, BQ) emerging as key contributors to water quality variance. These findings suggest that leachate migration, coupled with *in situ* chemical transformations, is the dominant source of phenolic pollution, with variations across locations emphasizing the need for site-specific waste management strategies. Pearson correlation analysis indicates that phenolic transformations in rural aquifers are governed by geochemical processes, while urban groundwater is largely influenced by continuous anthropogenic inputs. Given the paucity of regulatory benchmarks for HQ, RE, BQ, and BPA, there is an urgent need to establish comprehensive monitoring frameworks and enforce permissible limits for these phenolic compounds in drinking water while raising public awareness about groundwater contamination. This study provides a robust baseline for future toxicological, hydrogeological, and policy-oriented research, highlighting the critical need for integrated waste–aquifer management in rapidly urbanizing regions of sub-Saharan Africa.

## Author contributions

Esther A. Nnamani: conceptualization, resources, investigation, methodology, writing – original draft, formal analysis, writing – review editing, Ajibola A. Bayode: writing review & editing,^63,64^ Moses O. Alfred: writing review & editing, Brigitte Helmreich: funding, writing review & editing, Emmanuel I. Unuabonah: funding acquisition, conceptualization, supervision, resources, project administration, methodology, writing – review & editing, Martins O. Omorogie: conceptualization, funding acquisition, writing – review & editing, supervision, resources.

## Conflicts of interest

The authors declare that there is no conflict of interest to declare in this manuscript.

## Supplementary Material

RA-016-D5RA07962D-s001

## Data Availability

An anonymized metadata set has been deposited in the data repository. Supplementary information (SI) is available. See DOI: https://doi.org/10.1039/d5ra07962d.
