# Supplementary material for: Spatiotemporal distribution and risk assessment of bisphenol A and structurally related phenolic compounds in groundwater around the vicinity of municipal dumpsites in Southwestern Nigeria
Source: RSC Adv. 2026 Feb 13;16(10):8781–96. doi: 10.1039/d5ra07962d (PMC12903074; doi:10.1039/d5ra07962d)
Supplement: RA-016-D5RA07962D-s001 [file RA-016-D5RA07962D-s001.pdf]

Supplementary Information (SI) for RSC Advances.
This journal is © The Royal Society of Chemistry 2026

SUPPORTING INFORMATION (SI)

Spatiotemporal Distribution and Risk Assessment of Bisphenol A and Structurally Related Phenolic Compounds in Groundwater around the Vicinity of Municipal Dumpsites in Southwestern Nigeria

Esther A. Nnamani[1,2], Ajibola A. Bayode[2], Moses O. Alfred[1,2], Brigitte Helmreich[3,], Emmanuel I. Unuabonah[1,2], Martins O. Omorogie[1,2,3,‡]

[1]*African Centre of Excellence for Water and Environmental Research (ACEWATER), Redeemer's University, 232101, Ede, Nigeria*

[2]*Department of Chemical Sciences, Redeemer's University, 232101, Ede, Nigeria*

[3]*Chair of Urban Water Systems Engineering, School of Engineering and Design, Technical University of Munich, Am Coulombwall 3, D-85748, Garching, Germany*

[‡]**Corresponding Author:** mo.omorogie@tum.de, omorogiem@run.edu.ng, dromorogiemoon@gmail.com

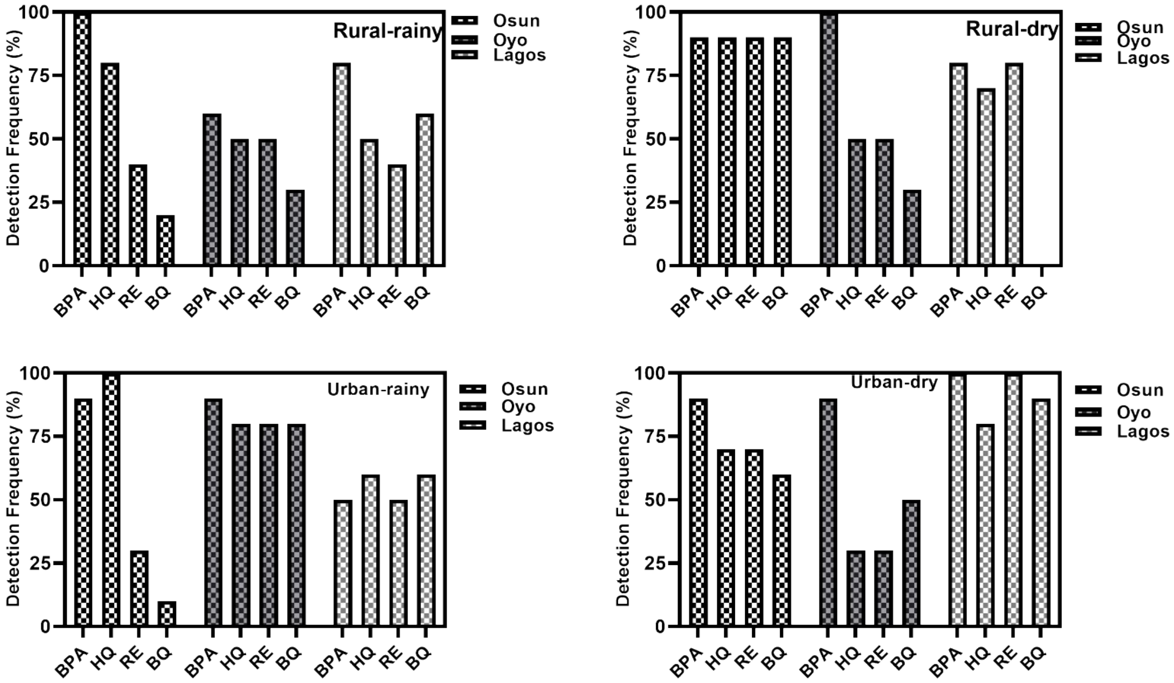

Figure S1: Seasonal Detection Frequencies for Bisphenol A (BPA), Hydroquinone (HQ), Resorcinol (RE), and Benzoquinone (BQ) in groundwater sources from rural and urban areas of Osun, Oyo, and Lagos States.

Table SI 1: Physicochemical Properties of the Targeted Analyte of the Study

PC	Chemical structure	Water solubility (25 °C) (mg/L)	pKa	Log K_{ow}	Ref.
Bisphenol A	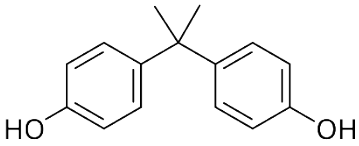	300	10.29	3.32	[1]
Hydroquinone	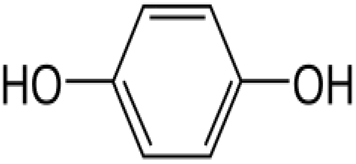	72,000	9.9	0.59	[2]
1,4-benzoquinone	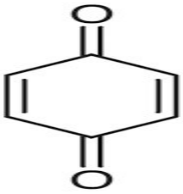	10,000	N/A	0.20	[3]
Resorcinol	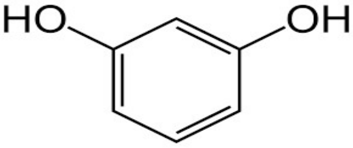	717,000	9.15	0.80	[4]

Table SI 2: Geographical Coordinates of Sampling Points

State/Setting	Location	Groundwater Type	Coordinates	Sample ID
Osun (R)	Ede	Borehole	7°45′37″ N, 4°26′29″ E	ED 1
		Borehole	7°44′16″ N, 4°25′58″ E	ED 2
		Borehole	7°43′54″ N, 4°25′24″ E	ED 3
		Well	7°41′39″ N, 4°27′13″ E	ED 4
		Well	7°43′14″ N, 4°26′51″ E	ED 5
		Borehole	7°41′51″ N, 4°27′18″ E	ED 6
		Well	7°41′57″ N, 4°27′32″ E	ED 7
		Well	7°41′47″ N, 4°27′10″ E	ED 8
		Borehole	7°41′44″ N, 4°27′28″ E	ED 9
		Borehole	7°41′50″ N, 4°27′15″ E	ED 10
Osun (U)	Osogbo	Well	7°46′14″ N, 4°32′17″ E	OS 1
		Borehole	7°46′54″ N, 4°32′58″ E	OS 2

		Borehole	7°47′28″ N, 4°32′14″ E	OS 3
		Well	7°48′30″ N, 4°34′58″ E	OS 4
		Borehole	7°46′28″ N, 4°31′59″ E	OS 5
		Borehole	7°46′04″ N, 4°32′31″ E	OS 6
		Well	7°47′14″ N, 4°32′58″ E	OS 7
		Borehole	7°45′22″ N, 4°33′09″ E	OS 8
		Borehole	7°45′26″ N, 4°32′59″ E	OS 9
		Borehole	7°46′12″ N, 4°32′32″ E	OS 10
Oyo (R)	Asejire	Well	7°21′33″ N, 4°09′32″ E	AS 1
		Well	7°20′59″ N, 4°07′35″ E	AS 2
		Well	7°21′02″ N, 4°08′03″ E	AS 3
		Borehole	7°20′59″ N, 4°07′57″ E	AS 4
		Borehole	7°21′26″ N, 4°08′06″ E	AS 5
		Well	7°21′20″ N, 4°08′04″ E	AS 6
		Well	7°21′08″ N, 4°08′26″ E	AS 7
		Borehole	7°20′49″ N, 4°08′01″ E	AS 8
		Well	7°21′31″ N, 4°07′20″ E	AS 9
		Well	7°21′23″ N, 4°07′35″ E	AS 10
Oyo (U)	Ibadan	Borehole	7°23′22″ N, 3°54′32″ E	IB 1
		Borehole	7°23′18″ N, 3°54′36″ E	IB 2
		Well	7°23′13″ N, 3°54′31″ E	IB 3
		Well	7°23′23″ N, 3°54′38″ E	IB 4
		Borehole	7°23′34″ N, 3°54′44″ E	IB 5
		Well	7°23′26″ N, 3°54′09″ E	IB 6
		Borehole	7°23′17″ N, 3°54′20″ E	IB 7
		Borehole	7°23′19″ N, 3°54′21″ E	IB 8
		Borehole	7°22′53″ N, 3°53′53″ E	IB 9
		Well	7°22′40″ N, 3°53′50″ E	IB 10
Lagos (U)	Ojota	Borehole	6°35′26″ N, 3°22′39″ E	OJ 1
		Borehole	6°35′41″ N, 3°22′21″ E	OJ 2
		Borehole	6°35′25″ N, 3°22′25″ E	OJ 3
		Borehole	6°35′21″ N, 3°22′26″ E	OJ 4
		Well	6°35′22″ N, 3°22′27″ E	OJ 5

		Borehole	6°35′32″ N, 3°22′22″ E	OJ 6
		Well	6°35′44″ N, 3°22′29″ E	OJ 7
		Borehole	6°35′30″ N, 3°22′28″ E	OJ 8
		Well	6°35′15″ N, 3°22′25″ E	OJ 9
		Borehole	6°35′13″ N, 3°22′24″ E	OJ 10
Lagos (R)	Badagry	Well	6°27′11″ N, 2°52′28″ E	BAD 1
		Well	6°27′09″ N, 2°52′33″ E	BAD 2
		Borehole	6°27′07″ N, 2°52′38″ E	BAD 3
		Well	6°27′05″ N, 2°52′43″ E	BAD 4
		Borehole	6°27′03″ N, 2°52′48″ E	BAD 5
		Borehole	6°27′01″ N, 2°52′53″ E	BAD 6
		Borehole	6°26′59″ N, 2°52′58″ E	BAD 7
		Well	6°26′57″ N, 2°53′03″ E	BAD 8
		Borehole	6°26′55″ N, 2°53′08″ E	BAD 9
		Borehole	6°26′53″ N, 2°53′13″ E	BAD 10

N.B: R= rural; U=Urban

Table SI 3: Seasonal and Geographical Mean Concentration (mg/L)

Compound	Location	Rural Rainy (Mean ± SD)	Rural Dry (Mean ± SD)	Urban Rainy (Mean ± SD)	Rural Dry (Mean ± SD)
BPA	Osun	1.06 ± 0.90	19.16 ± 6.47	1.88 ± 1.38	20.90 ± 9.54
	Oyo	4.82 ± 2.04	6.36 ± 1.00	6.65 ± 3.74	3.99 ± 2.05
	Lagos	4.50 ± 2.84	14.42 ± 4.18	11.10 ± 13.69	11.90 ± 5.67
HQ	Osun	7.26 ± 6.78	4.16 ± 3.33	4.50 ± 4.81	3.72 ± 5.12
	Oyo	2.27 ± 1.37	11.93 ± 1.51	1.35 ± 1.39	8.15 ± 6.61
	Lagos	2.18 ± 1.70	3.74 ± 4.71	6.65 ± 9.87	3.85 ± 2.73
RE	Osun	2.30 ± 0.42	6.40 ± 5.28	2.61 ± 2.56	2.07 ± 1.90
	Oyo	0.73 ± 0.75	4.54 ± 0.86	1.87 ± 1.93	6.63 ± 2.90
	Lagos	3.06 ± 2.00	3.70 ± 6.45	6.97 ± 10.60	20.70 ±14.14
BQ	Osun	1.61 ± 1.23	5.45 ± 1.90	0.83 (1 value)	1.31 ± 2.31
	Oyo	4.06 ± 6.65	9.30 ± 4.24	2.20 ± 2.64	6.54 ± 3.64
	Lagos	3.90 ± 3.20	-	3.20 ± 1.83	4.50 ± 3.22

Table SI 4: Statistical Summary of the Studied PCs in Groundwater

Location	Season	Parameters (mg/L)	BPA	HQ	RE	BQ
Osun (R)	Rainy	Median	0.79	8.08	2.14	1.61
		Min-Max	0.22-3.02	0.00-18.20	0.00-2.87	0.74-2.48
Osun (R)	Dry	Median	19.29	4.07	4.57	6.10
		Min-Max	5.94-29.03	0.00-7.80	0.79-16.43	1.23-7.14
Osun (U)	Rainy	Median	1.57	4.05	1.24	0.83 (*)
		Min-Max	0.00-4.71	0.09-13.53	0.00-5.57	-
Osun (U)	Dry	Median	25.50	2.52	1.44	0.36
		Min-Max	0.00-38.79	0.00-14.99	0.00-5.86	0.00-6.01
Oyo (R)	Rainy	Median	4.06	1.97	1.05	1.22
		Min-Max	0.00-7.65	0.00-3.21	0.00-10.33	0.54-17.48
Oyo (R)	Dry	Median	6.45	11.60	4.27	7.14
		Min-Max	4.59-7.48	0.00-13.68	0.00-5.96	6.55-14.17
Oyo (U)	Rainy	Median	7.11	0.63	1.82	1.19
		Min-Max	0.00-12.15	0.00-3.69	0.00-6.11	0.00-3.03

Oyo (U)	Dry	Median	3.08	9.71	7.93	8.27
		Min-Max	0.00-7.11	0.00-13.84	0.00-8.66	0.00-9.61
Lagos (R)	Rainy	Median	5.65	2.89	3.83	3.52
		Min-Max	0.00-8.50	0.00-4.01	0.00-4.45	0.00-8.08
Lagos (R)	Dry	Median	16.01	1.97	0.96	**
		Min-Max	0.00-18.23	0.00-13.68	0.00-30.66	**
Lagos (U)	Rainy	Median	5.45	2.60	2.43	3.44
		Min-Max	0.00-35.58	0.00-24.13	0.00-4.10	0.00-43.61
Lagos (U)	Dry	Median	10.15	3.91	19.98	4.22
		Min-Max	0.00-20.75	0.00-7.93	0.00-47.37	0.00-24.71

Footnote: * means only one (1) reported concentration; ** means 0% detection frequency; ND was treated as zero; Rural (R); Urban (U)

Table SI 5: Summary of the Comparative Statistics across the Sampled States

**State**	**Location**	**Compound–Season Effect (Rainy vs Dry × Compounds)**	**Interaction (Location × Season-Compound)**	**Significance Summary**
Osun	F = 6.50, *p* = 0.012 (significant)	F = 4.48, *p*< 0.001 (highly significant)	F = 0.89, *p* = 0.519 (not significant)	Location and season significantly influenced the levels of compounds.
Oyo	F = 0.34, *p* = 0.563 (Not significant)	F = 3.80, *p* = 0.0008 (Significant)	F = 1.76, *p* = 0.101 (not significant)	Seasonal variation was present; spatial effects were minimal.
Lagos	F = 12.82, *p* = 0.0005 (Highly significant)	F = 9.39, *p*< 0.000001 (Highly significant)	F = 4.48, *p* = 0.0002 (Significant)	Strong influence of both spatial and seasonal factors, including their interaction.

Table SI 6: Global Comparative Data onTargeted PC Compounds in Groundwater

Location	Year	Analyte	Reported levels (mg/L)	Ref.
China	2019	BPA	ND-0.0349	[5]
China	2018	BPA	0.000067–0.000118	[6]
China	2024	BPA	0-0.00016	[7]
Poland	2025	BPA	2.58-5.90	[8]
Nigeria	2023	BPA	ND	[9]
Nigeria	2019	BPA	0.008-0.081	[10]
Nigeria	2023	HQ	0.005-0.053	[11]

Footnote: ND-not detected

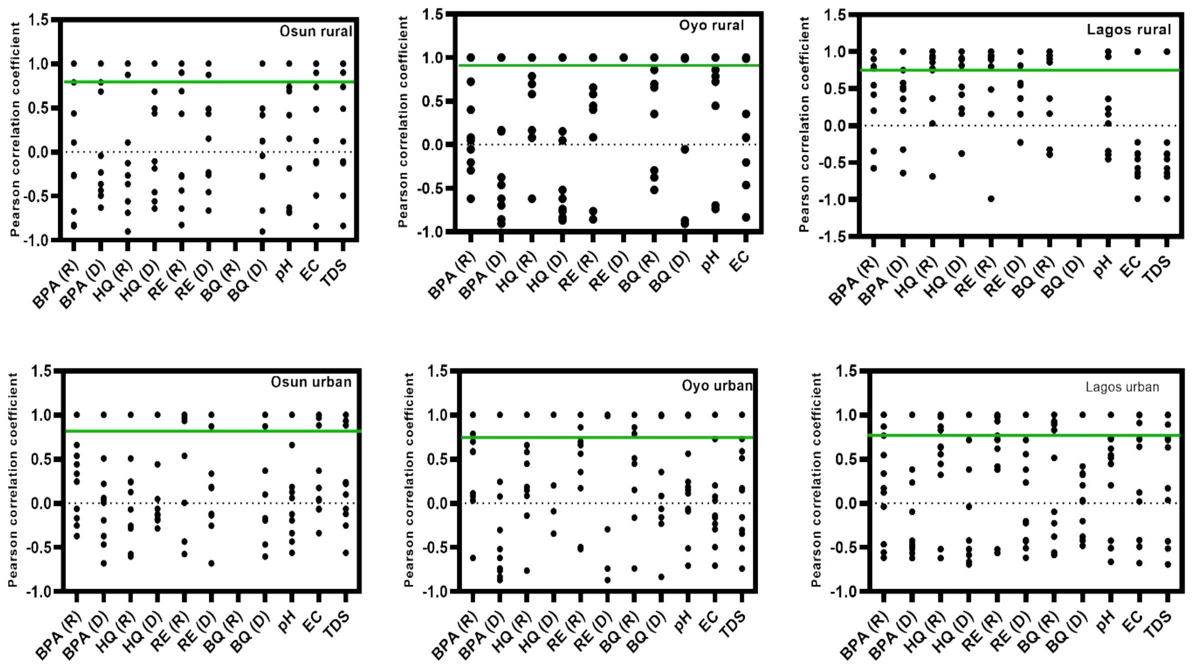

Figure S2: Pearson correlation analysis of phenolic compounds and physicochemical parameters in groundwater from Osun, Oyo, and Lagos.

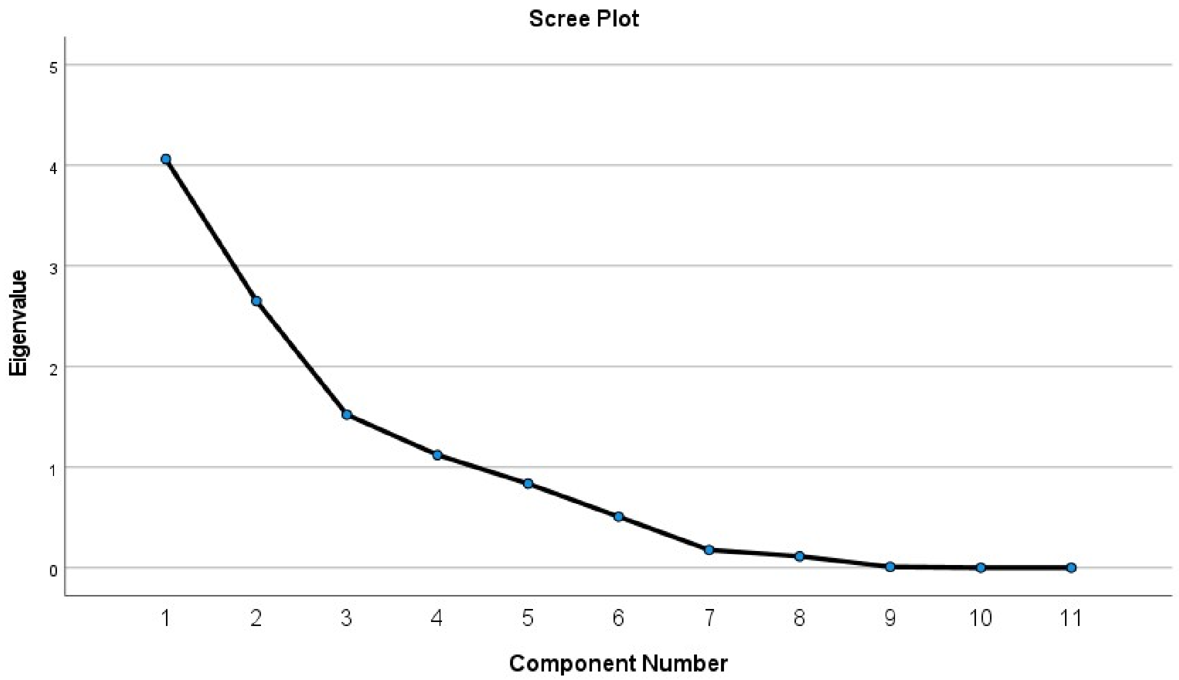
Scree Plot
Eigenvalue
Component Number
0
1
2
3
4
5
1
2
3
4
5
6
7
8
9
10
11

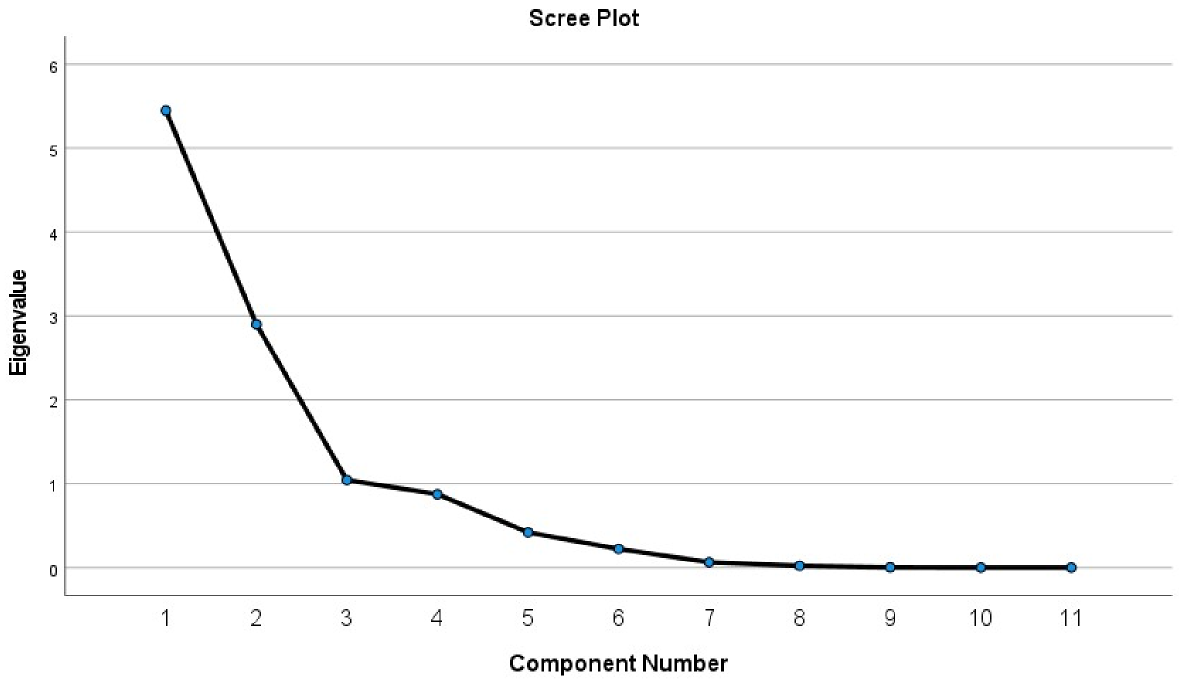
Scree Plot
Eigenvalue
Component Number
0
1
2
3
4
5
6
1
2
3
4
5
6
7
8
9
10
11

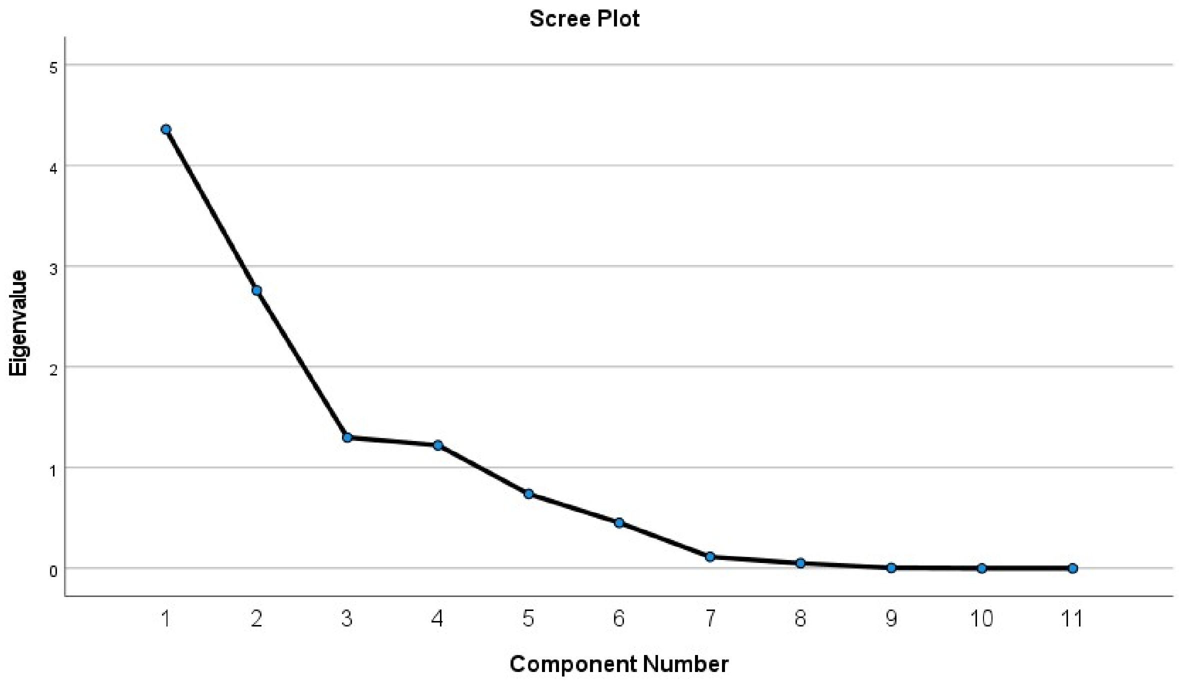

Figure S3: Representative Scree Plots from the Multivariate analysis

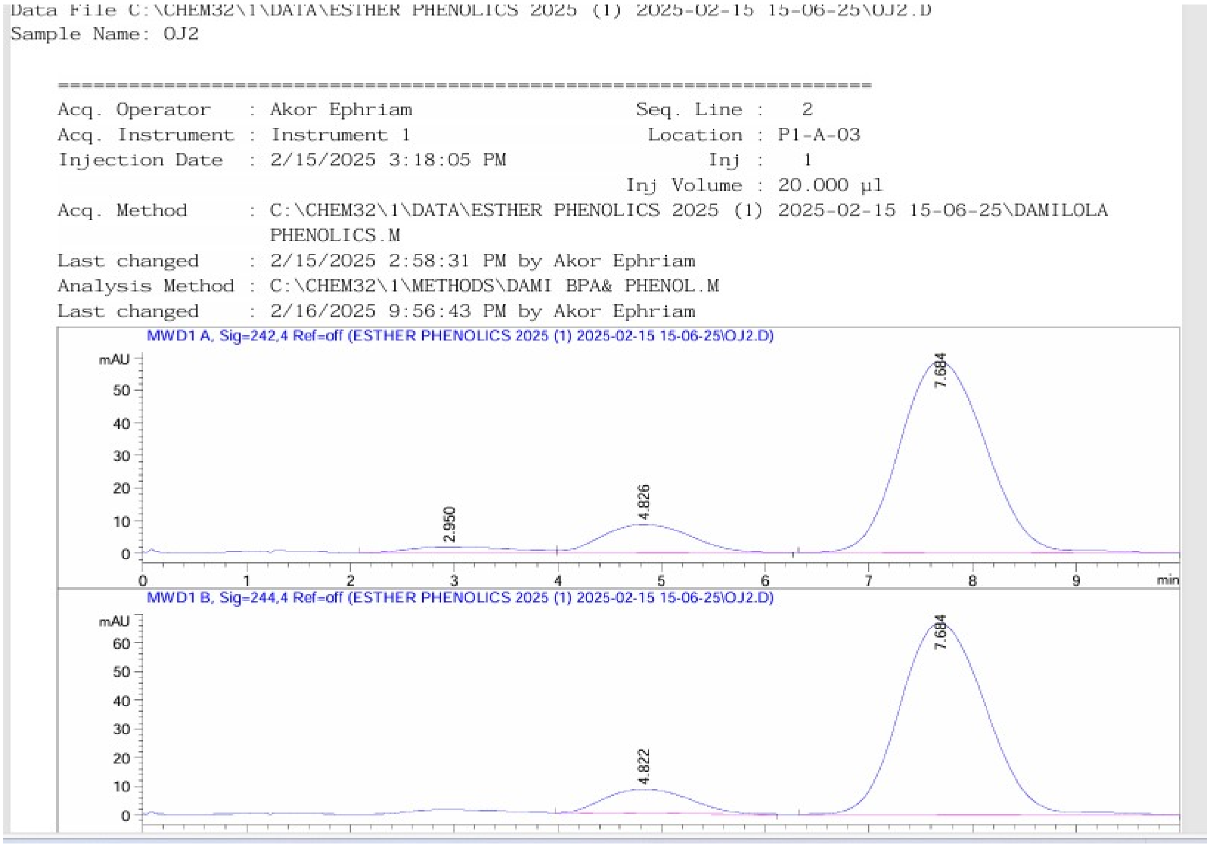
Data File C:\CHEM32\1\DATA\ESTHER PHENOLICS 2025 (1) 2025-02-15 15-06-25\OJ2.D
Sample Name: OJ2

===

Acq. Operator : Akor Ephriam Seq. Line : 2
Acq. Instrument : Instrument 1 Location : P1-A-03
Injection Date : 2/15/2025 3:18:05 PM Inj : 1
Inj Volume : 20.000 µl
Acq. Method : C:\CHEM32\1\DATA\ESTHER PHENOLICS 2025 (1) 2025-02-15 15-06-25\DAMILOLA PHENOLICS.M
Last changed : 2/15/2025 2:58:31 PM by Akor Ephriam
Analysis Method : C:\CHEM32\1\METHODS\DAMI BPA& PHENOL.M
Last changed : 2/16/2025 9:56:43 PM by Akor Ephriam

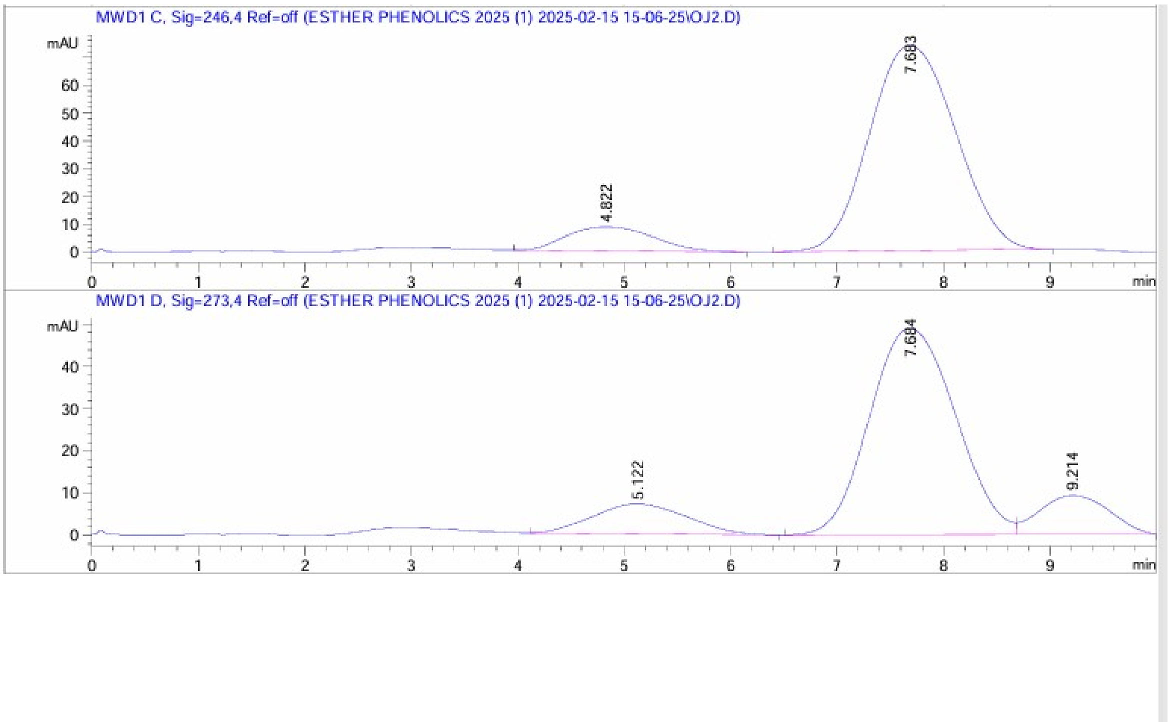

Data File C:\CHEM32\1\DATA\ESTHER PHENOLICS 2025 (1) 2025-02-15 15-06-25\OJ2.D
Sample Name: OJ2

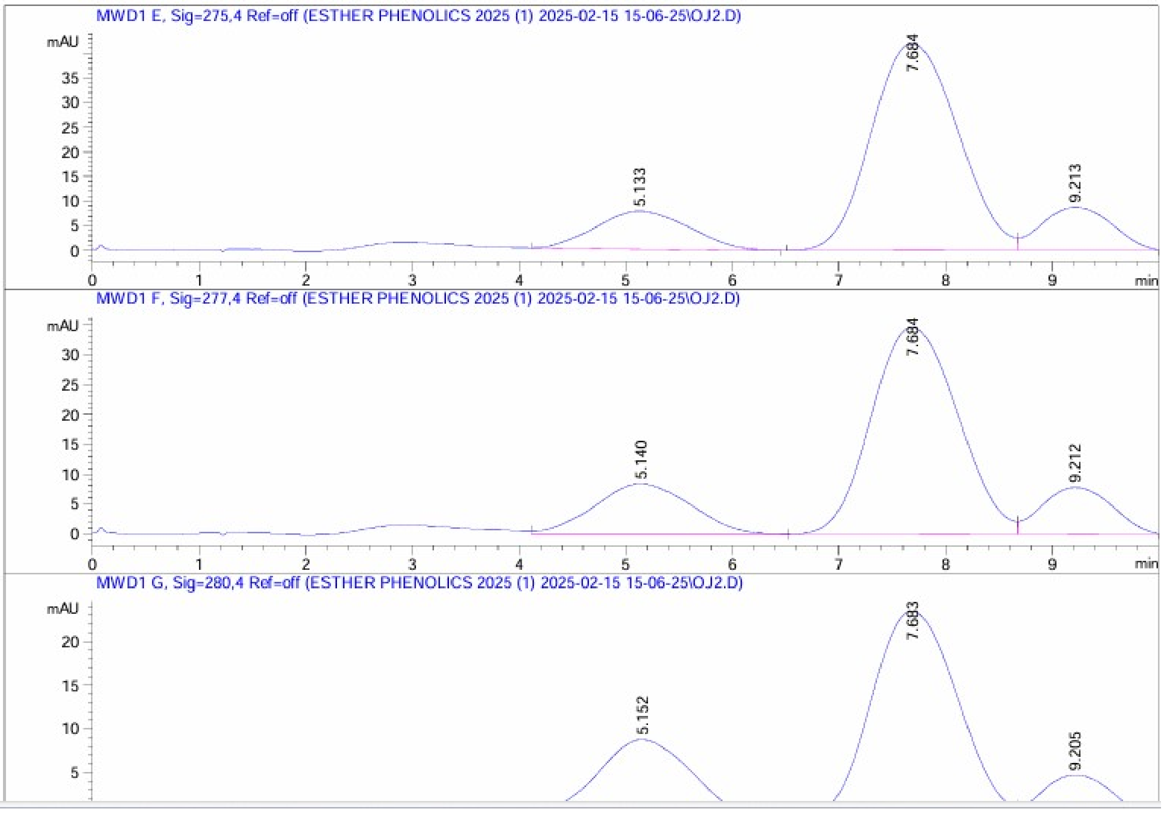

Data File C:\CHEM32\1\DATA\ESTHER PHENOLICS 2025 (1) 2025-02-15 15-06-25\OJ2.D
Sample Name: OJ2

Signal 1: MWD1 A, Sig=242,4 Ref=off

Peak #	RetTime [min]	Type	Width [min]	Area [mAU*s]	Height [mAU]	Area %
1	2.950	BV	0.8170	122.39460	1.77388	3.0658
2	4.826	VB	0.7976	506.84491	8.59921	12.6957
3	7.684	BBA	0.9161	3363.00952	58.72576	84.2385
Totals :				3992.24903	69.09885	

Signal 2: MWD1 B, Sig=244,4 Ref=off

Peak #	RetTime [min]	Type	Width [min]	Area [mAU*s]	Height [mAU]	Area %
1	4.822	BB	0.7686	475.99622	8.40619	11.0957
2	7.684	BBA	0.9127	3813.92480	66.54444	88.9043
Totals :				4289.92102	74.95063	

Signal 3: MWD1 C, Sig=246,4 Ref=off

Peak #	RetTime [min]	Type	Width [min]	Area [mAU*s]	Height [mAU]	Area %

Signal 3: MWD1 C, Sig=246,4 Ref=off

Peak #	RetTime [min]	Type	Width [min]	Area [mAU*s]	Height [mAU]	Area %
1	4.822	BB	0.8271	482.01343	8.48031	10.4712
2	7.683	BB	0.8911	4121.19824	73.61546	89.5288
Totals :				4603.21167	82.09576	

Signal 4: MWD1 D, Sig=273,4 Ref=off

Peak #	RetTime [min]	Type	Width [min]	Area [mAU*s]	Height [mAU]	Area %
1	5.122	BB	0.8312	436.05441	7.15267	12.0767
2	7.684	BV	0.8884	2748.55444	49.00406	76.1220
3	9.214	VBA	0.7177	426.11166	9.31461	11.8013
Totals :				3610.72052	65.47134	

Signal 5: MWD1 E, Sig=275,4 Ref=off

Signal 5: MWD1 E, Sig=275,4 Ref=off

Peak #	RetTime [min]	Type	Width [min]	Area [mAU*s]	Height [mAU]	Area %
1	5.133	BB	0.8353	467.87930	7.69606	14.5471
2	7.684	BV	0.8997	2353.14063	41.99511	73.1628
3	9.213	VBA	0.7173	395.28616	8.64795	12.2901
Totals :				3216.30609	58.33912	

Signal 6: MWD1 F, Sig=277,4 Ref=off

Peak #	RetTime [min]	Type	Width [min]	Area [mAU*s]	Height [mAU]	Area %
1	5.140	VB	0.8677	534.45142	8.47578	18.8964
2	7.684	BV	0.8932	1939.95093	34.64640	68.5902
3	9.212	VBA	0.7225	353.91718	7.75363	12.5133

Signal 7: MWD1 G, Sig=280,4 Ref=off

Peak #	RetTime [min]	Type	Width [min]	Area [mAU*s]	Height [mAU]	Area %
1	5.152	BB	0.7687	515.50903	8.60785	25.0006
2	7.683	BV	0.8912	1321.62708	23.60577	64.0949
3	9.205	VBA	0.5688	224.84856	4.92375	10.9045
Totals :				2061.98466	37.13738	

Signal 8: MWD1 H, Sig=282,4 Ref=off

Peak #	RetTime [min]	Type	Width [min]	Area [mAU*s]	Height [mAU]	Area %
1	5.158	BB	0.8252	513.68829	8.64500	31.0290
2	7.677	BV	0.8458	1004.94690	17.93335	60.7031
3	9.210	VBA	0.5473	136.87772	2.99447	8.2680
Totals :				1655.51291	29.57281	

===

*** End of Report ***

Figure S4: Representative Chromatogram Details of the Studied Phenolic Compounds

Reference

1. J. L. Torres-García, M. Ahuactzin-Pérez, F. J. Fernández and V. C.-E. Diana, *Chemosphere*, 2022, **303**, 134940.
2. F. J. Enguita and A. L. Leitão, *BioMed research international*, 2013, **2013**, 542168.
3. F. Orsolya, S.-B. Erzsébet and H. Ottó, *Journal of Photochemistry and Photobiology A: Chemistry*, 2021, **407**, 113057.
4. L. Qiyu, I. Fahrin, A. Zaki Uddin, L. Xiaobo, D. Dilip, Z. Mark, D. G. Daniel, H. William and Y. Hui, *Chemosphere*, 2021, **280**, 130730.
5. Z. Haifeng, Z. Yangping, L. Jiabao and Y. Min, *Science of The Total Environment*, 2019, **655**, 607-613.
6. R.-X. Li, C.-M. Wang, J.-k. Cao, W.-X. Cao, Q. Xu and J. Li, *International Journal of Environmental Analytical Chemistry*, 2018, **98**, 921-937.
7. N. Zhang, Z. Zhang, C. Li, J. Yue, Y. Su, W. Cheng, S. Sun, X. Chen, D. Shi and B. Liu, *Water*, 2024, **16**, 2575.
8. Ś. Kinga and D. Dominika, *Journal of Hydrology*, 2025, **654**, 132829.
9. T. N. Adebesin, S. A. Lateef, E. O. Oloruntoba and M. Adejumo, *Journal of Water and Health*, 2023, **21**, 740-750.
10. O. Onyekwere, C. J. Okonkwo, A. B. Okoroafor and C. J. Okonkwo, *Ovidius University Annals of Chemistry*, 2019, **30**, 101-107.
11. O. B. Otitoju, M. O. Alfred, C. G. Olorunnisola, F. T. Aderinola, O. O. Ogunlaja, O. D. Olukanni, A. Ogunlaja, M. O. Omorogie and E. I. Unuabonah, *RSC advances*, 2024, **14**, 982-994.
